# Supplementary figures and images for: Predicting and elucidating the etiology of fatty liver disease: A machine learning modeling and validation study in the IMI DIRECT cohorts
Source: PLoS Med. 2020 Jun 19;17(6):e1003149. doi: 10.1371/journal.pmed.1003149 (PMC7304567; doi:10.1371/journal.pmed.1003149)

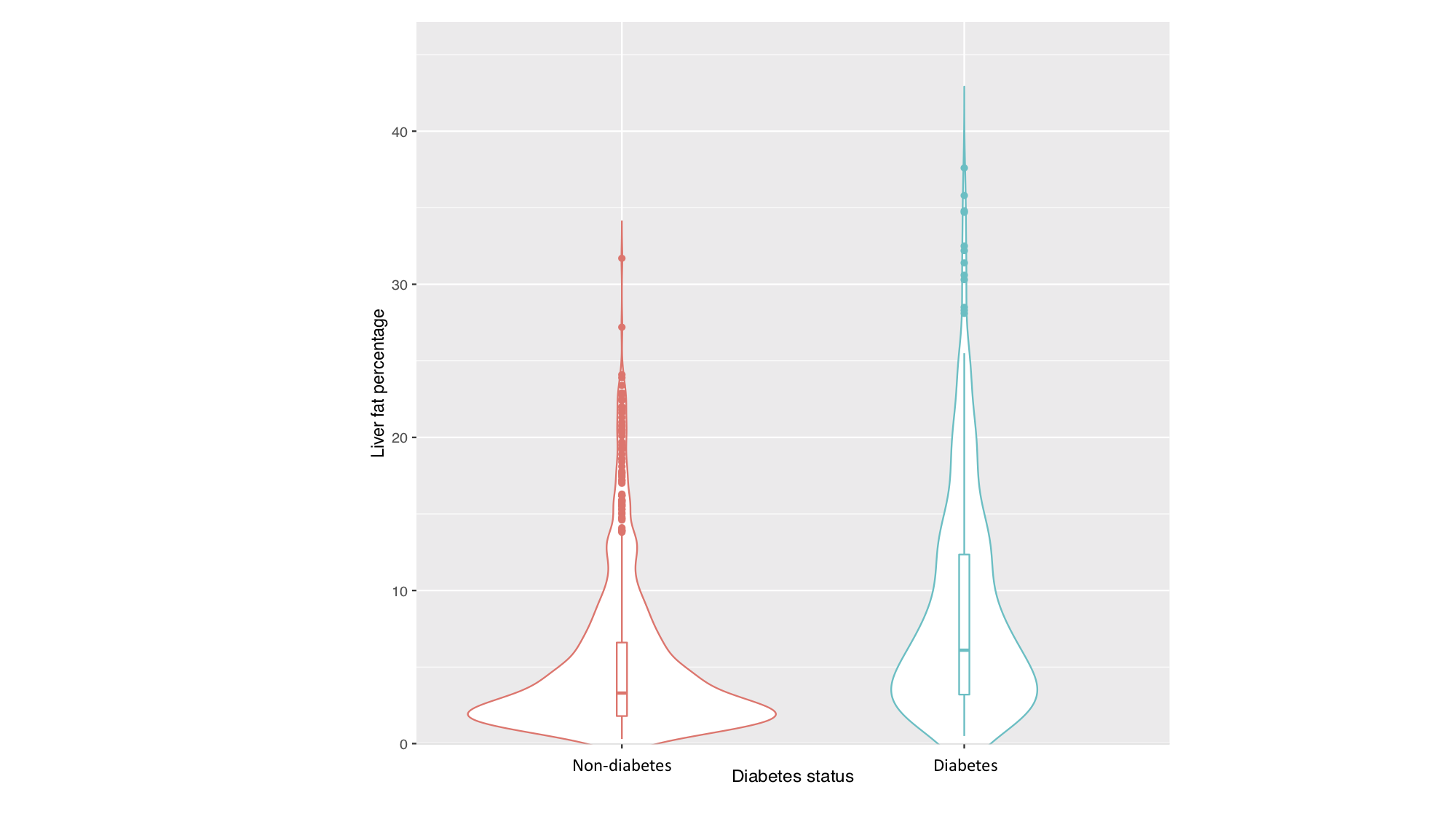

Supplement: S1 Fig — (TIFF) [file pmed.1003149.s001.tiff]

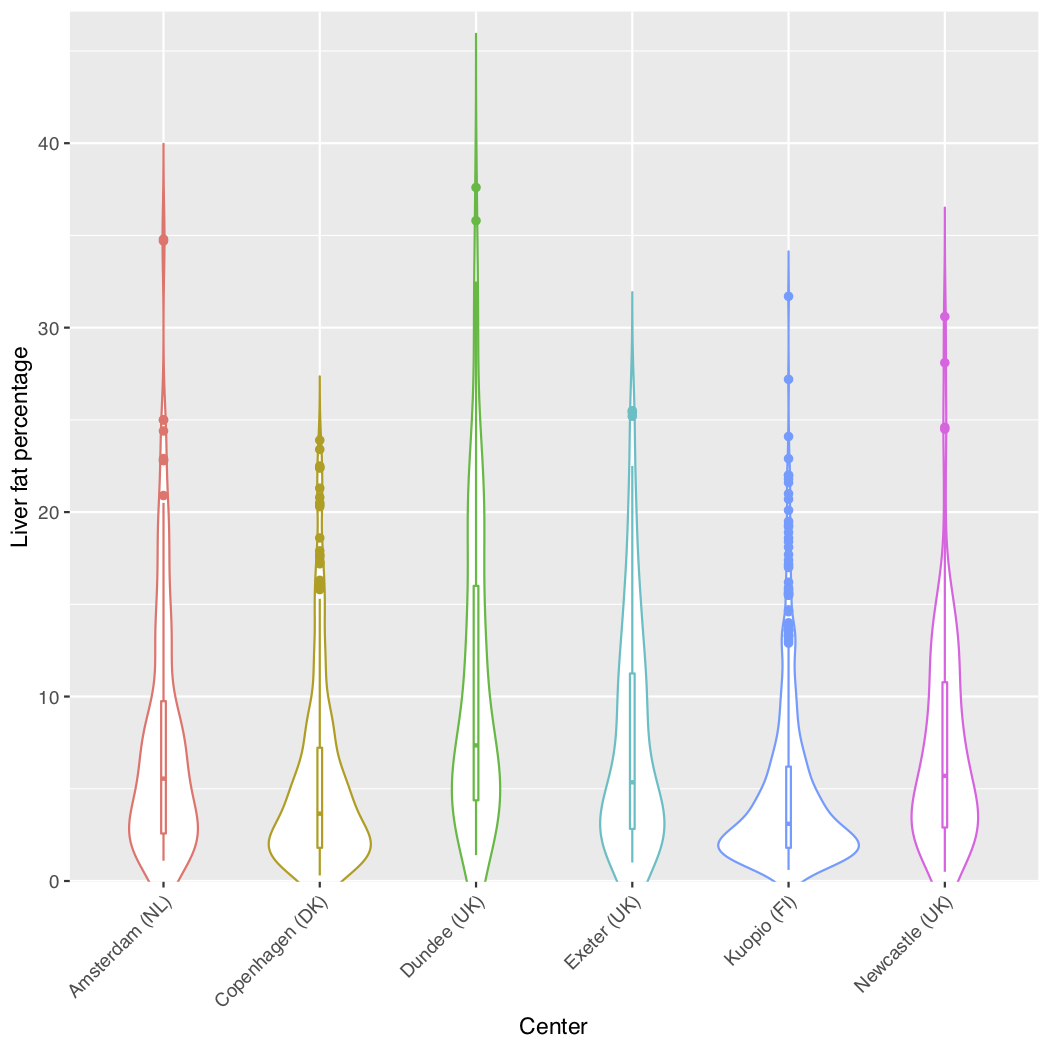

Supplement: S2 Fig — (TIFF) [file pmed.1003149.s002.tiff]

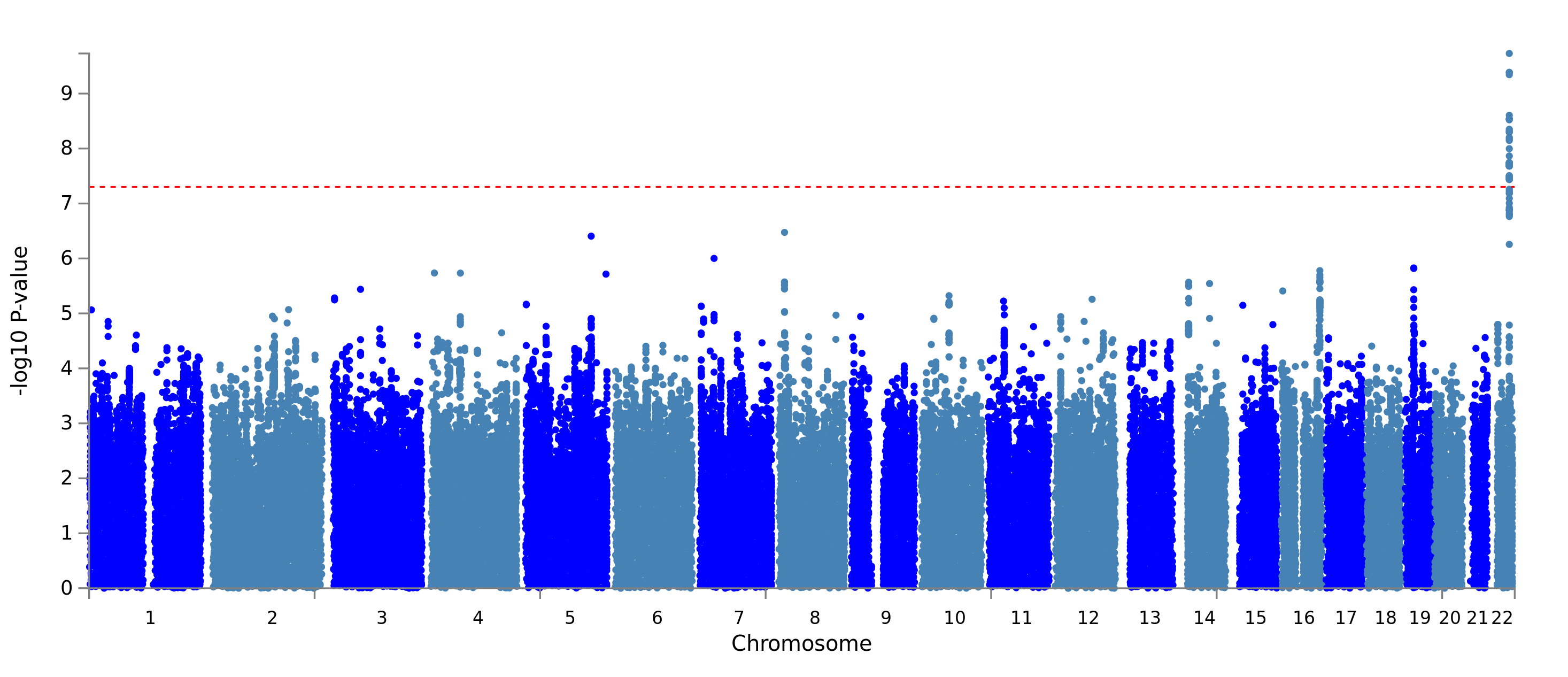

Supplement: S3 Fig — The chromosomal position is plotted on the x-axis, and the statistical significance of association for each SNP is plotted on the y-axis. Red line indicates genome-wide significance level (5 × 10^−8^). (TIFF) [file pmed.1003149.s003.tiff]

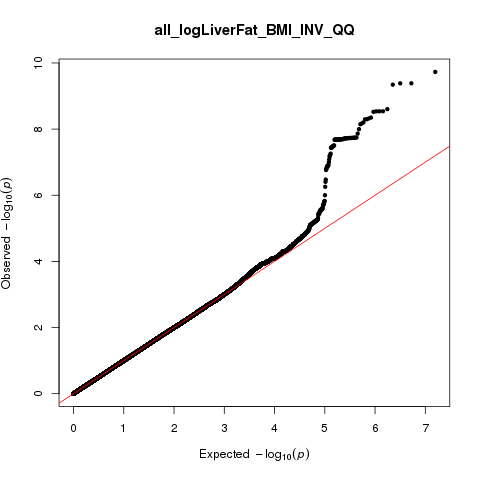

Supplement: S4 Fig — The x-axis illustrates the expected distribution of p-values from the association test across all SNPs, and the y-axis shows the observed p-values. (TIFF) [file pmed.1003149.s004.tiff]

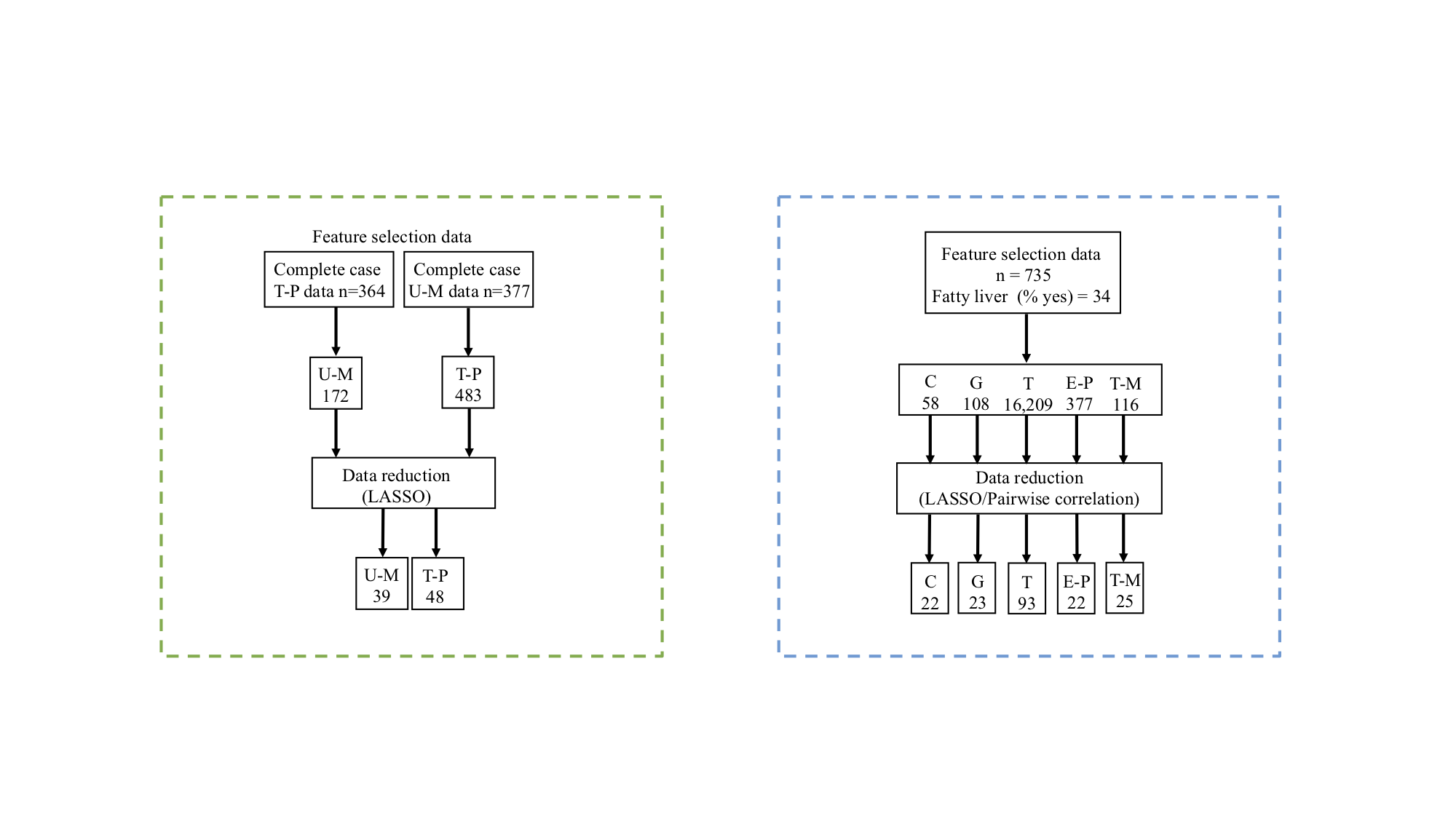

Supplement: S5 Fig — Models 4–14 (blue box); models 15–18 (green box). (TIFF) [file pmed.1003149.s005.tiff]

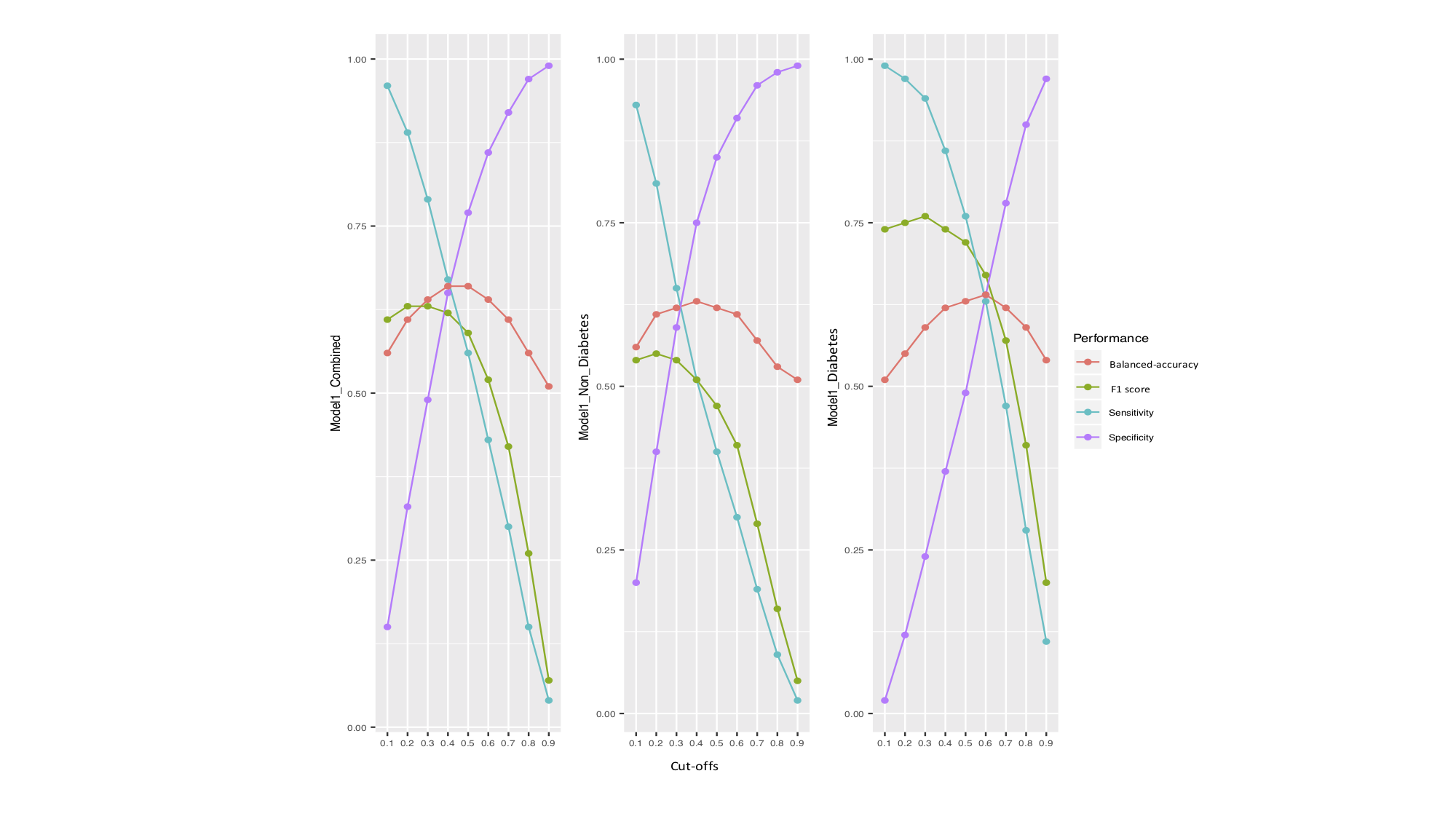

Supplement: S6 Fig — (TIFF) [file pmed.1003149.s006.tiff]

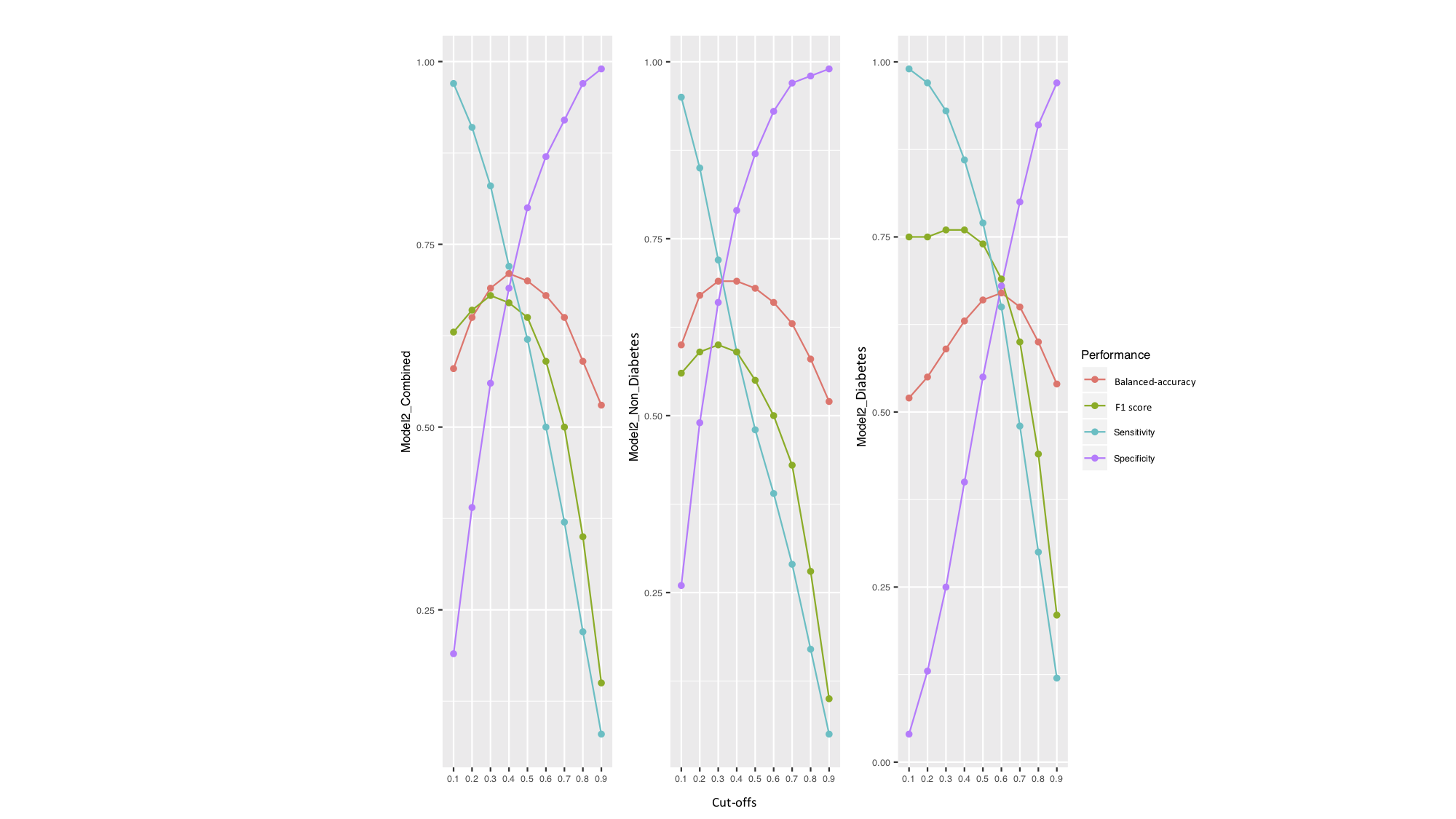

Supplement: S7 Fig — (TIFF) [file pmed.1003149.s007.tiff]

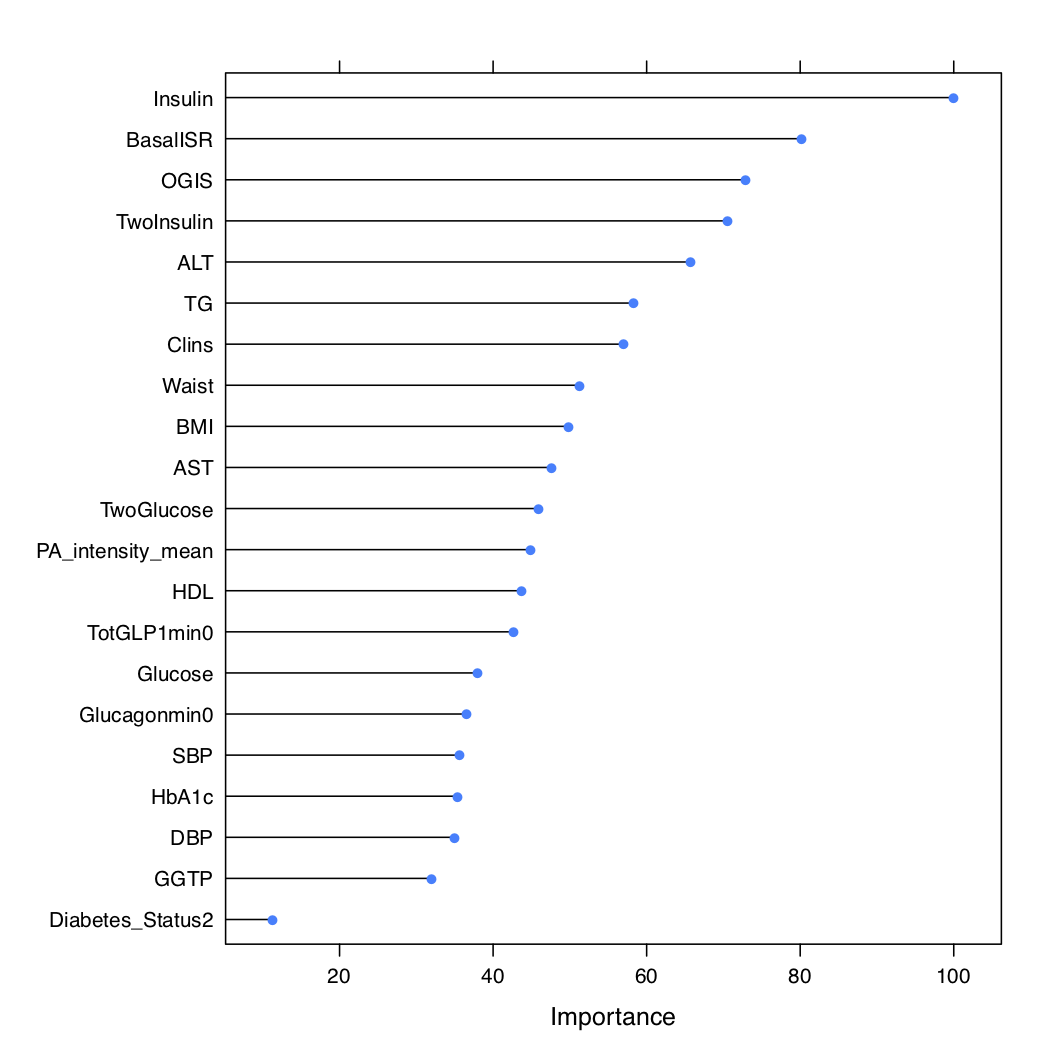

Supplement: S8 Fig — The y-axis shows the top 20 predictors in the model. The x-axis shows the variable importance, calculated using random forest analysis as the difference in prediction accuracy before and after the permutation for each variable scaled by the standard error. ALT, alanine transaminase; AST, aspartate transaminase; BasalISR, insulin secretion at the beginning of the OGTT/MMTT; BMI, body mass index; Clins, mean insulin clearance during the OGTT/MMTT calculated as (mean insulin secretion)/(mean insulin concentration); DBP, diastolic blood pressure; Diabetes_status2, non-diabetes/diabetes; GGTP, gamma-glutamyl transpeptidase; Glucagonmin0, fasting glucagon concentration; Glucose, fasting glucose from venous plasma samples; HbA1c, hemoglobin A1C; HDL, fasting high-density lipoprotein cholesterol; Insulin, fasting insulin from venous plasma samples; OGIS, oral glucose insulin sensitivity index according to the method of Mari et al. [24]; PA_intensity_mean, mean high-pass-filtered vector magnitude physical activity intensity; SBP, systolic blood pressure; TG, fasting triglycerides; TotGLP1min0, concentration of fasting total GLP-1 in plasma; TwoGlucose, 2-hour glucose after OGTT/MMTT; TwoInsulin, 2-hour insulin. (TIFF) [file pmed.1003149.s008.tiff]

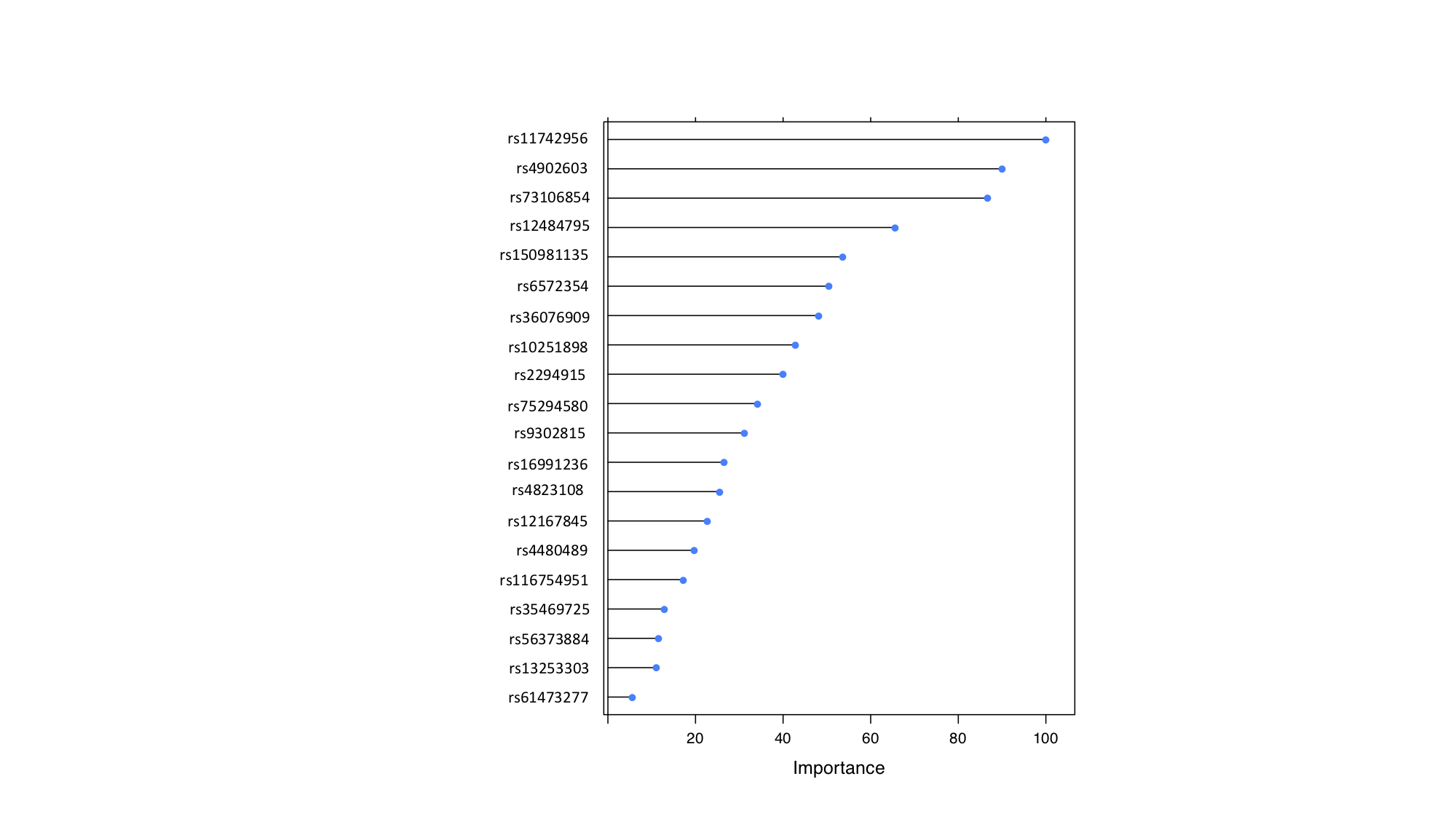

Supplement: S9 Fig — The y-axis shows the top 20 predictors in the model. The x-axis shows the variable importance, calculated using random forest analysis as the difference in prediction accuracy before and after the permutation for each variable scaled by the standard error. (TIFF) [file pmed.1003149.s009.tiff]

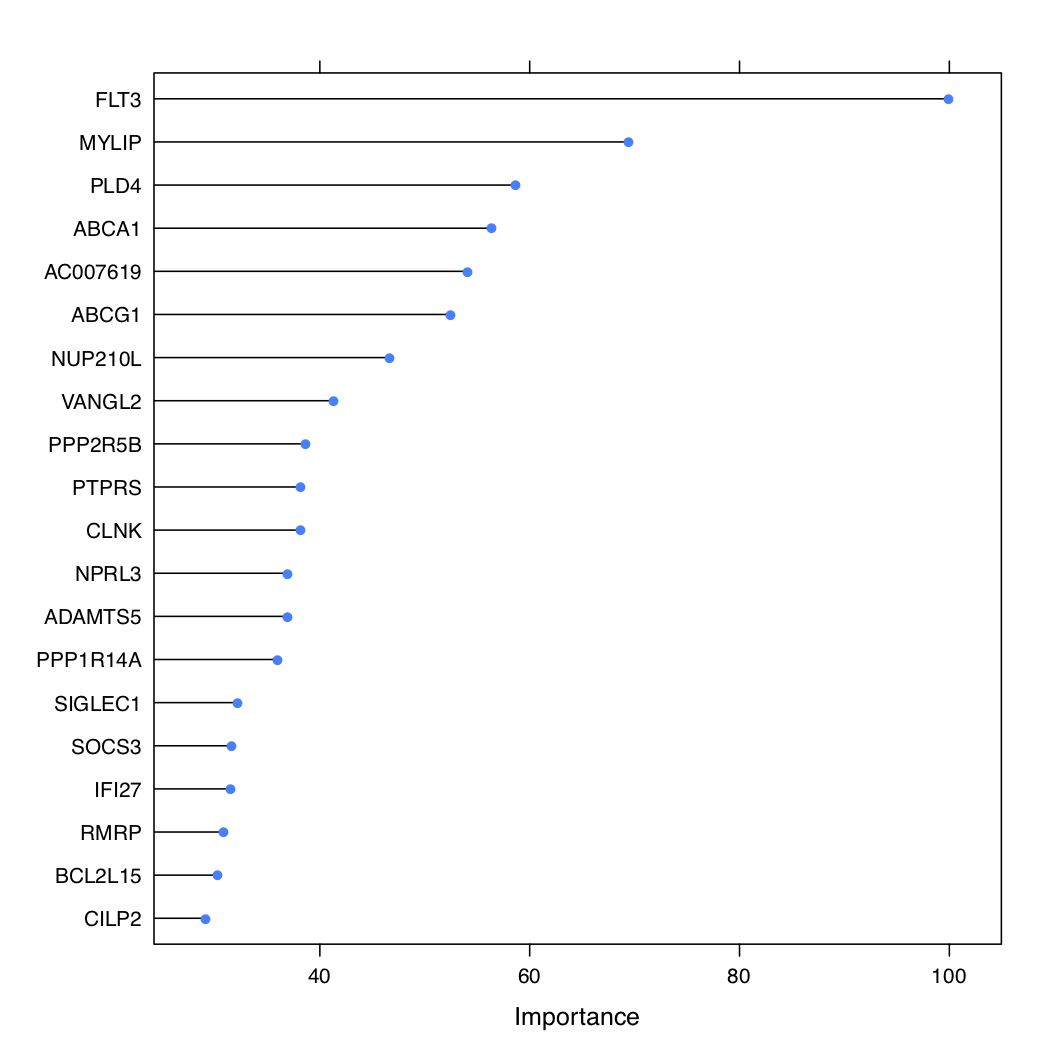

Supplement: S10 Fig — The y-axis shows the top 20 predictors in the model. The x-axis shows the variable importance, calculated using random forest analysis as the difference in prediction accuracy before and after the permutation for each variable scaled by the standard error. (TIFF) [file pmed.1003149.s010.tiff]

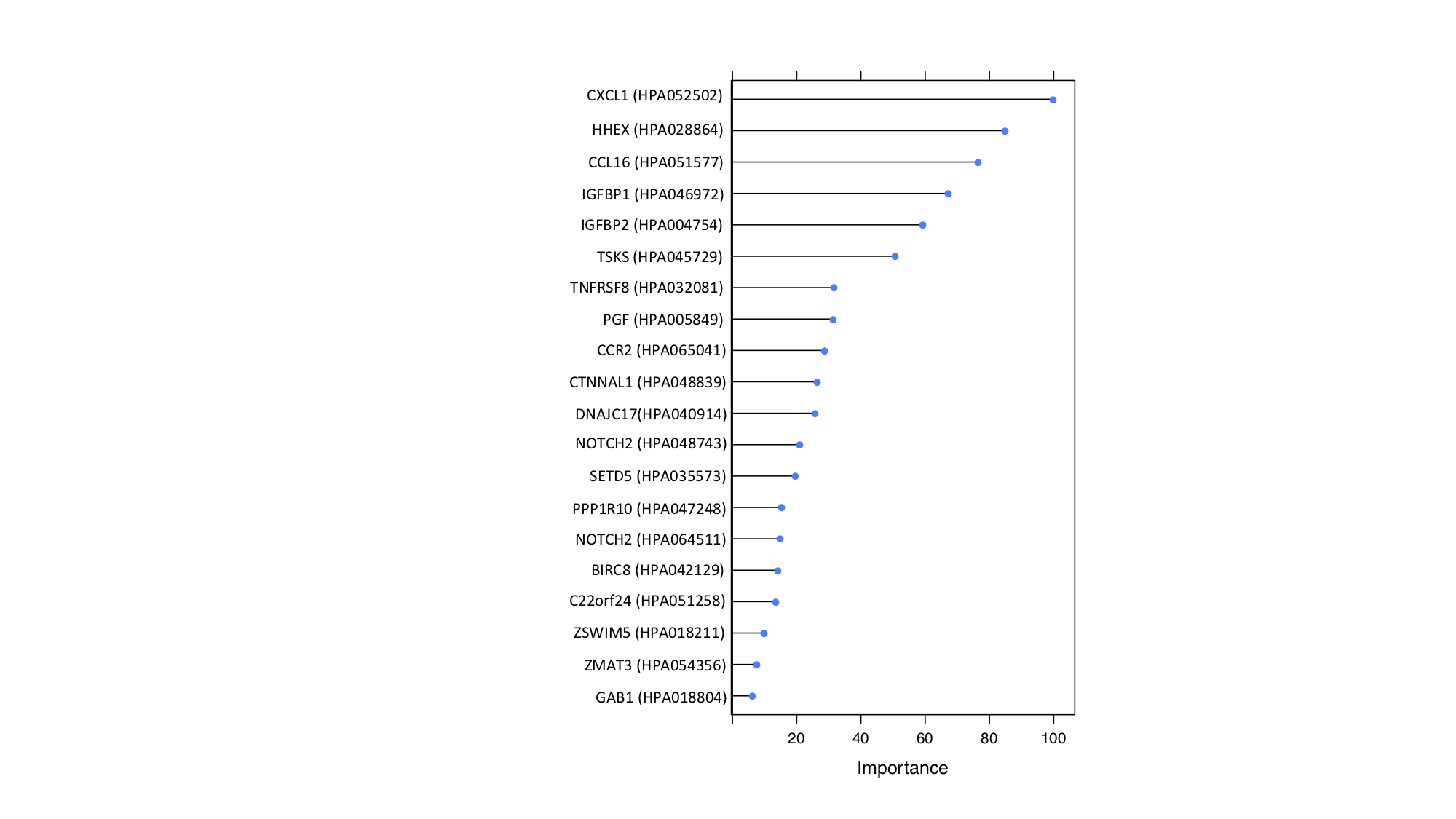

Supplement: S11 Fig — The y-axis shows the top 20 predictors in the model. The x-axis shows the variable importance, calculated using random forest as the difference in prediction accuracy before and after the permutation for each variable scaled by the standard error. (TIFF) [file pmed.1003149.s011.tiff]

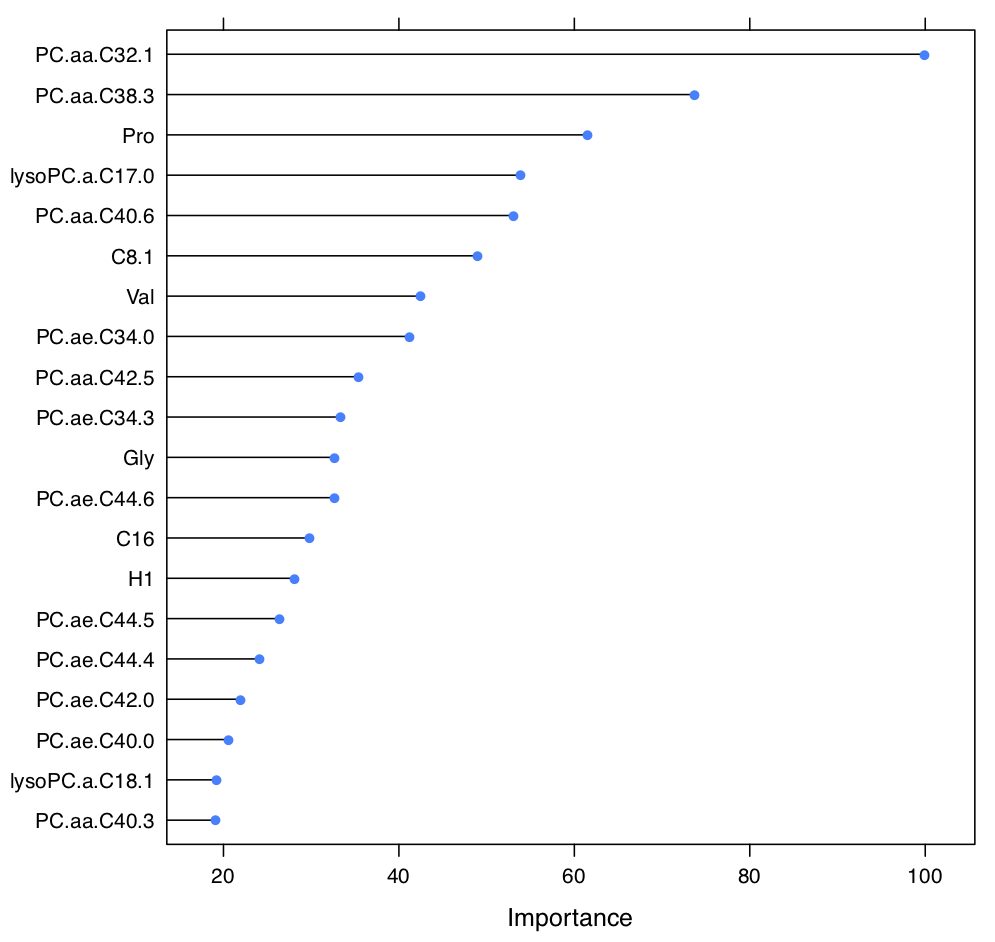

Supplement: S12 Fig — The y-axis shows the top 20 predictors in the model. The x-axis shows the variable importance, calculated using random forest analysis as the difference in prediction accuracy before and after the permutation for each variable scaled by the standard error. (TIFF) [file pmed.1003149.s012.tiff]

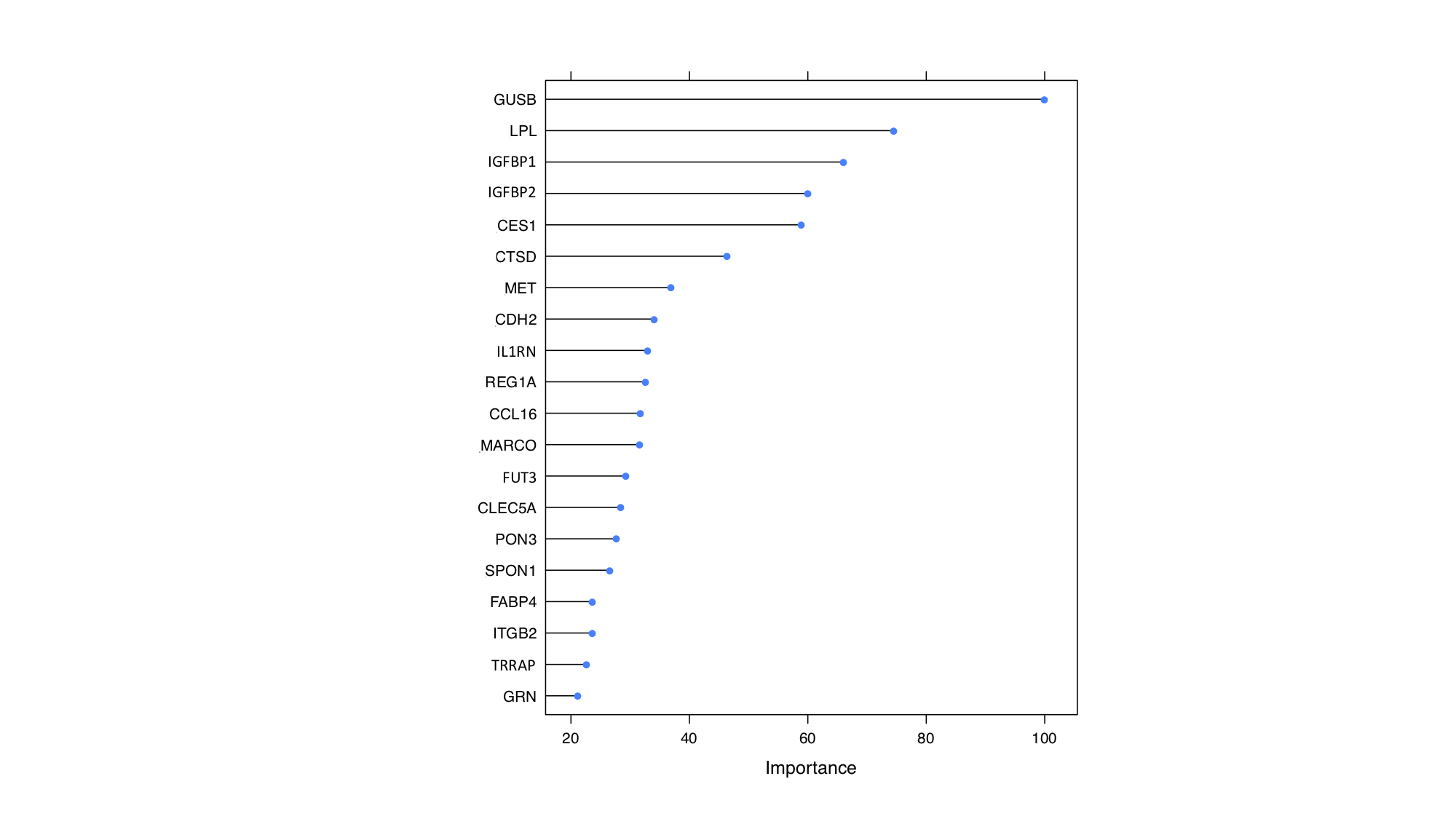

Supplement: S13 Fig — The y-axis shows the top 20 predictors in the model. The x-axis shows the variable importance, calculated using random forest analysis as the difference in prediction accuracy before and after the permutation for each variable scaled by the standard error. (TIFF) [file pmed.1003149.s013.tiff]

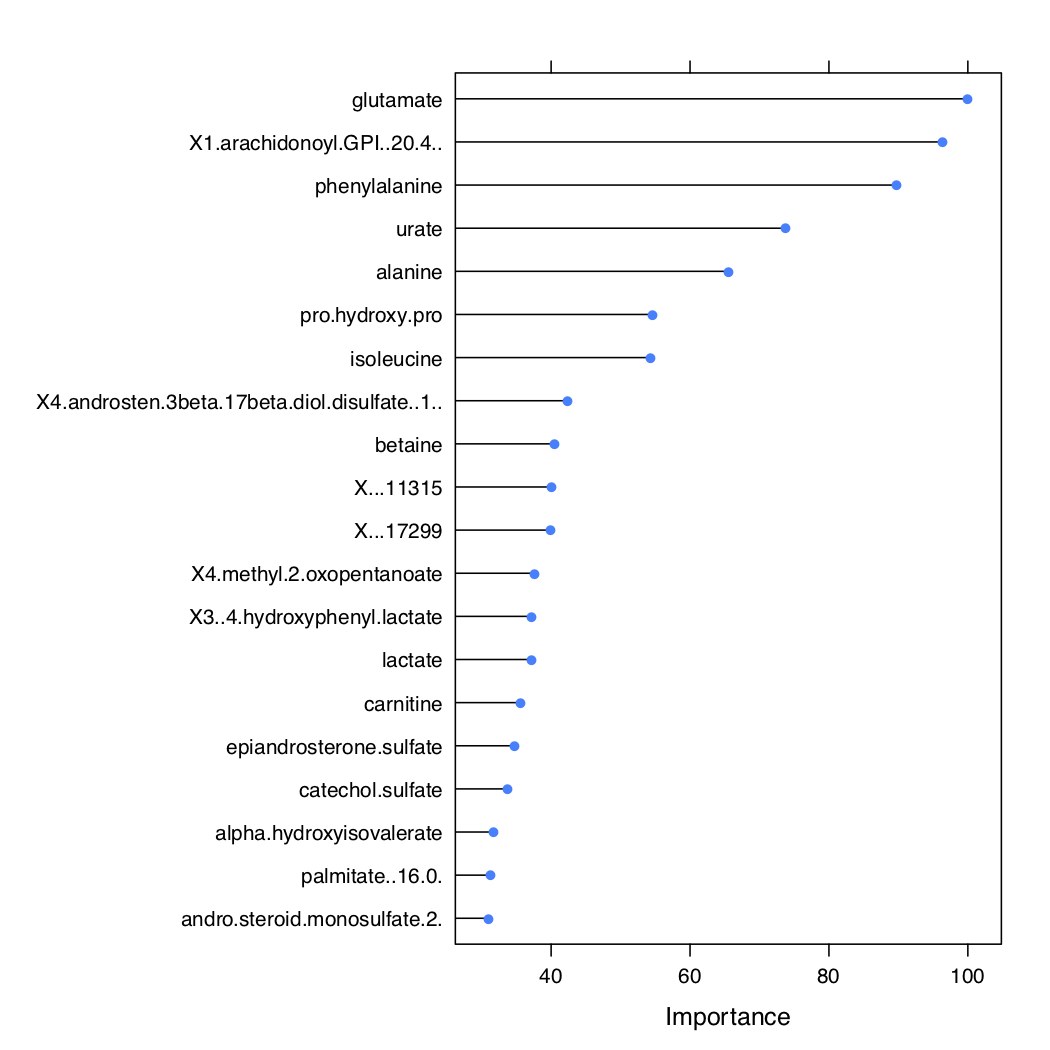

Supplement: S14 Fig — The y-axis shows the top 20 predictors in the model. The x-axis shows the variable importance, calculated using random forest analysis as the difference in prediction accuracy before and after the permutation for each variable scaled by the standard error. (TIFF) [file pmed.1003149.s014.tiff]

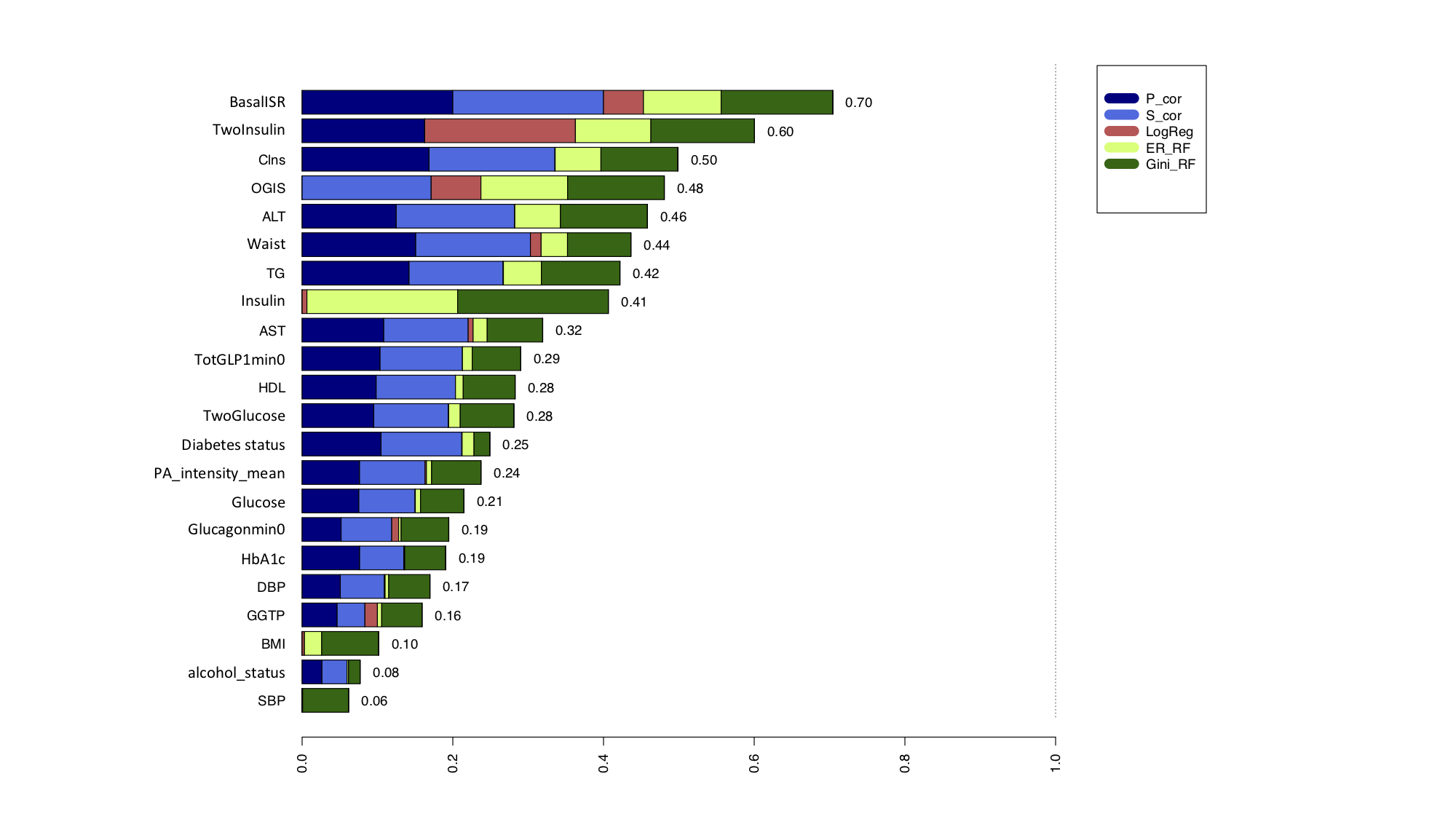

Supplement: S15 Fig — The y-axis shows the 22 clinical variables ordered by importance value. The x-axis shows the cumulative importance values, calculated via an ensemble of feature selection methods including Spearman’s rank correlation test (S_cor), Pearson’s product moment correlation test (P_cor), beta-values of logistic regression (LogReg), error-rate-based variable importance measure (ER_RF), and Gini-index-based variable importance measure (Gini_RF). ALT, alanine transaminase; AST, aspartate transaminase; BasalISR, insulin secretion at the beginning of the OGTT/MMTT; BMI, body mass index; Clins, mean insulin clearance during the OGTT/MMTT calculated as (mean insulin secretion)/(mean insulin concentration); DBP, diastolic blood pressure; Diabetes status, non-diabetes/diabetes; GGTP, gamma-glutamyl transpeptidase; Glucagonmin0, fasting glucagon concentration; Glucose, fasting glucose from venous plasma samples; HbA1c, hemoglobin A1C; HDL, fasting high-density lipoprotein cholesterol; Insulin, fasting insulin from venous plasma samples; OGIS, oral glucose insulin sensitivity index according to the method of Mari et al. [24]; PA_intensity_mean, mean high-pass-filtered vector magnitude physical activity intensity; SBP, systolic blood pressure; TG, fasting triglycerides; TotGLP1min0, concentration of fasting total GLP-1 in plasma; TwoGlucose, 2-hour glucose after OGTT/MMTT; TwoInsulin, 2-hour insulin. (TIF) [file pmed.1003149.s015.tif]

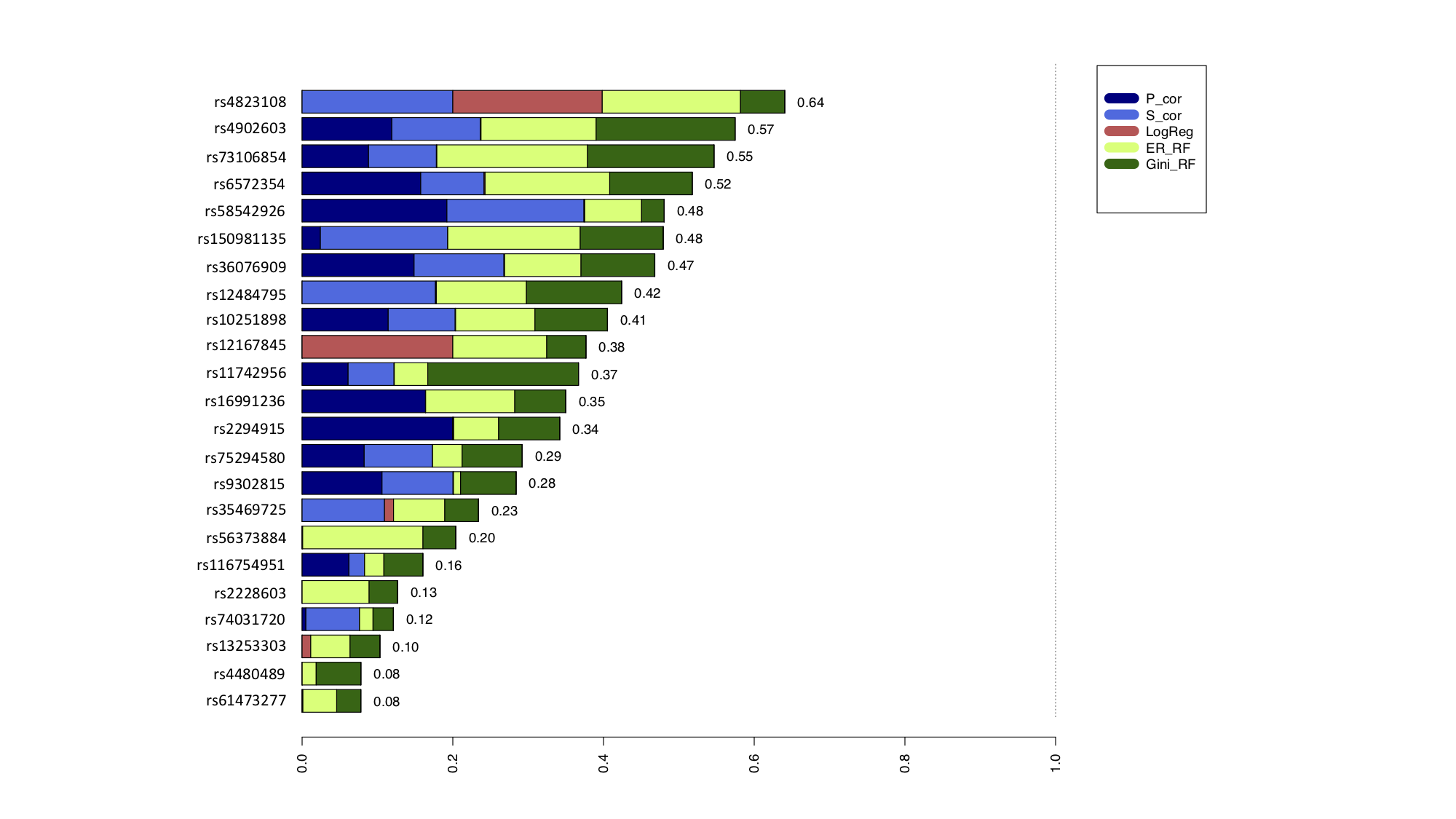

Supplement: S16 Fig — The y-axis shows the 23 genetic variables ordered by importance value. The x-axis shows the cumulative importance values, calculated via an ensemble of feature selection methods including Spearman’s rank correlation test (S_cor), Pearson’s product moment correlation test (P_cor), beta-values of logistic regression (LogReg), error-rate-based variable importance measure (ER_RF), and Gini-index-based variable importance measure (Gini_RF). (TIFF) [file pmed.1003149.s016.tiff]

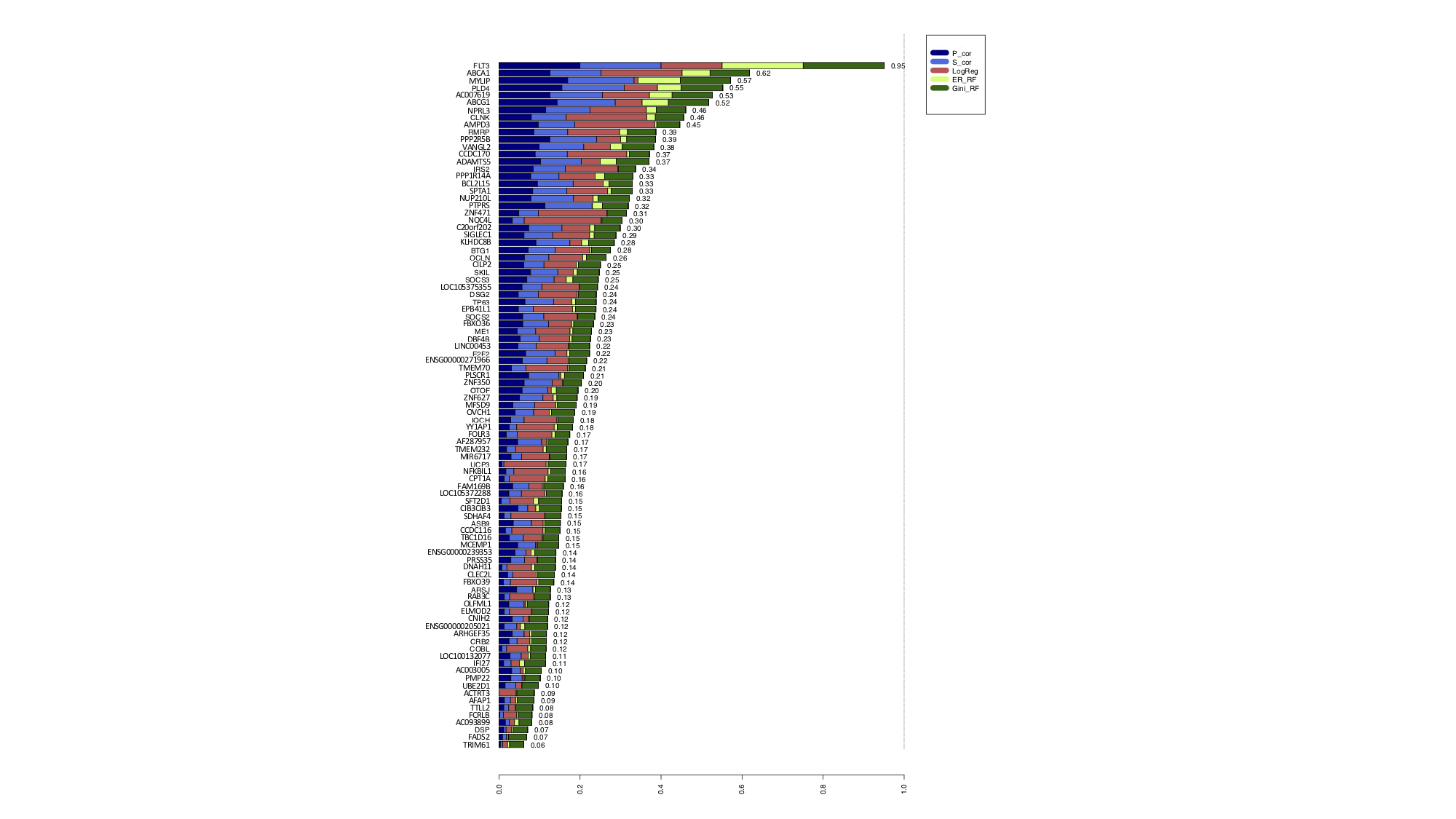

Supplement: S17 Fig — The y-axis shows the 93 transcriptomic variables ordered by importance value. The x-axis shows the cumulative importance values, calculated via an ensemble of feature selection methods including Spearman’s rank correlation test (S_cor), Pearson’s product moment correlation test (P_cor), beta-values of logistic regression (LogReg), error-rate-based variable importance measure (ER_RF), and Gini-index-based variable importance measure (Gini_RF). (TIFF) [file pmed.1003149.s017.tiff]

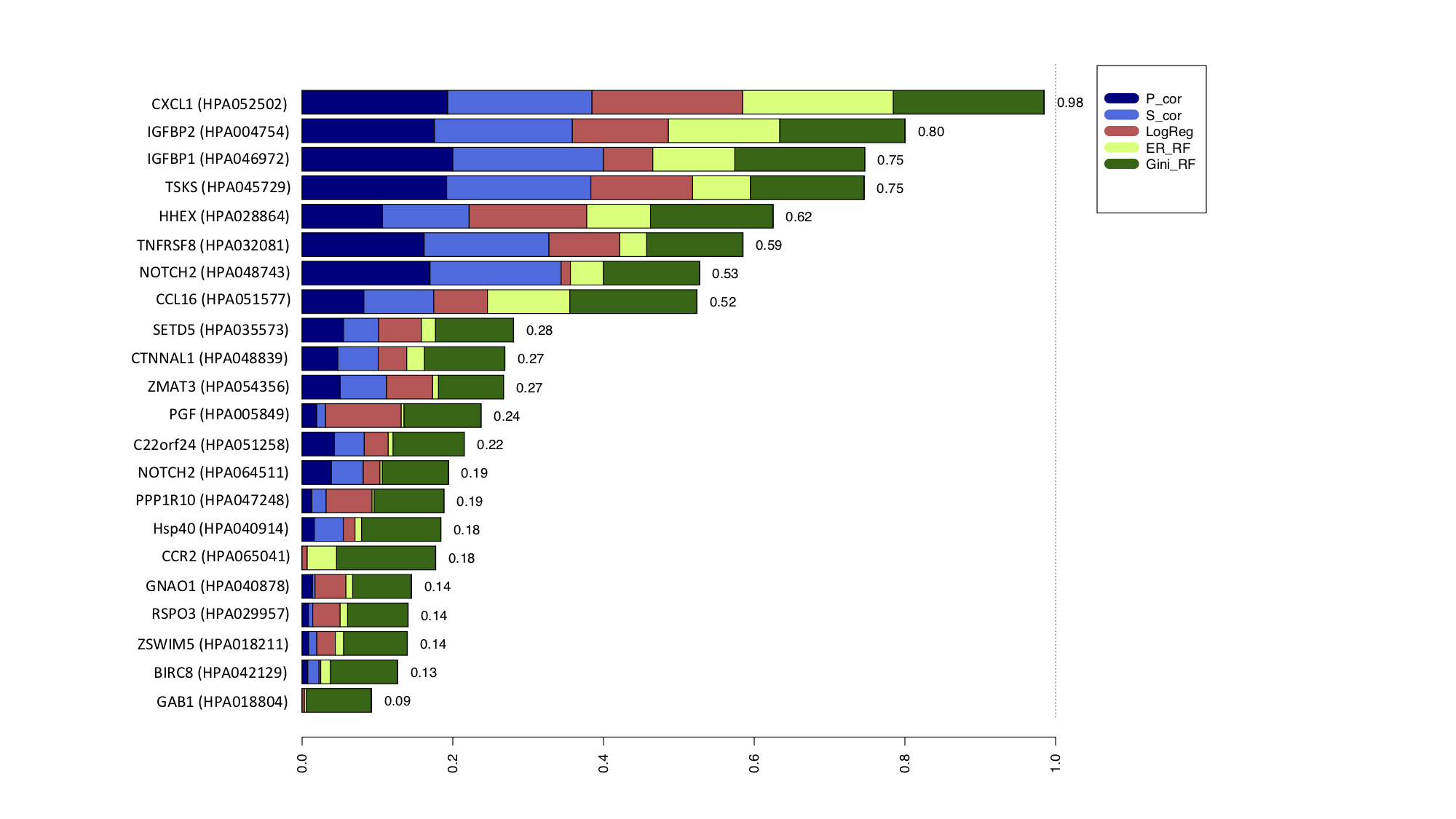

Supplement: S18 Fig — The y-axis shows the 22 exploratory proteomic variables ordered by importance value. The x-axis shows the cumulative importance values, calculated via an ensemble of feature selection methods including Spearman’s rank correlation test (S_cor), Pearson’s product moment correlation test (P_cor), beta-values of logistic regression (LogReg), error-rate-based variable importance measure (ER_RF), and Gini-index-based variable importance measure (Gini_RF). (TIFF) [file pmed.1003149.s018.tiff]

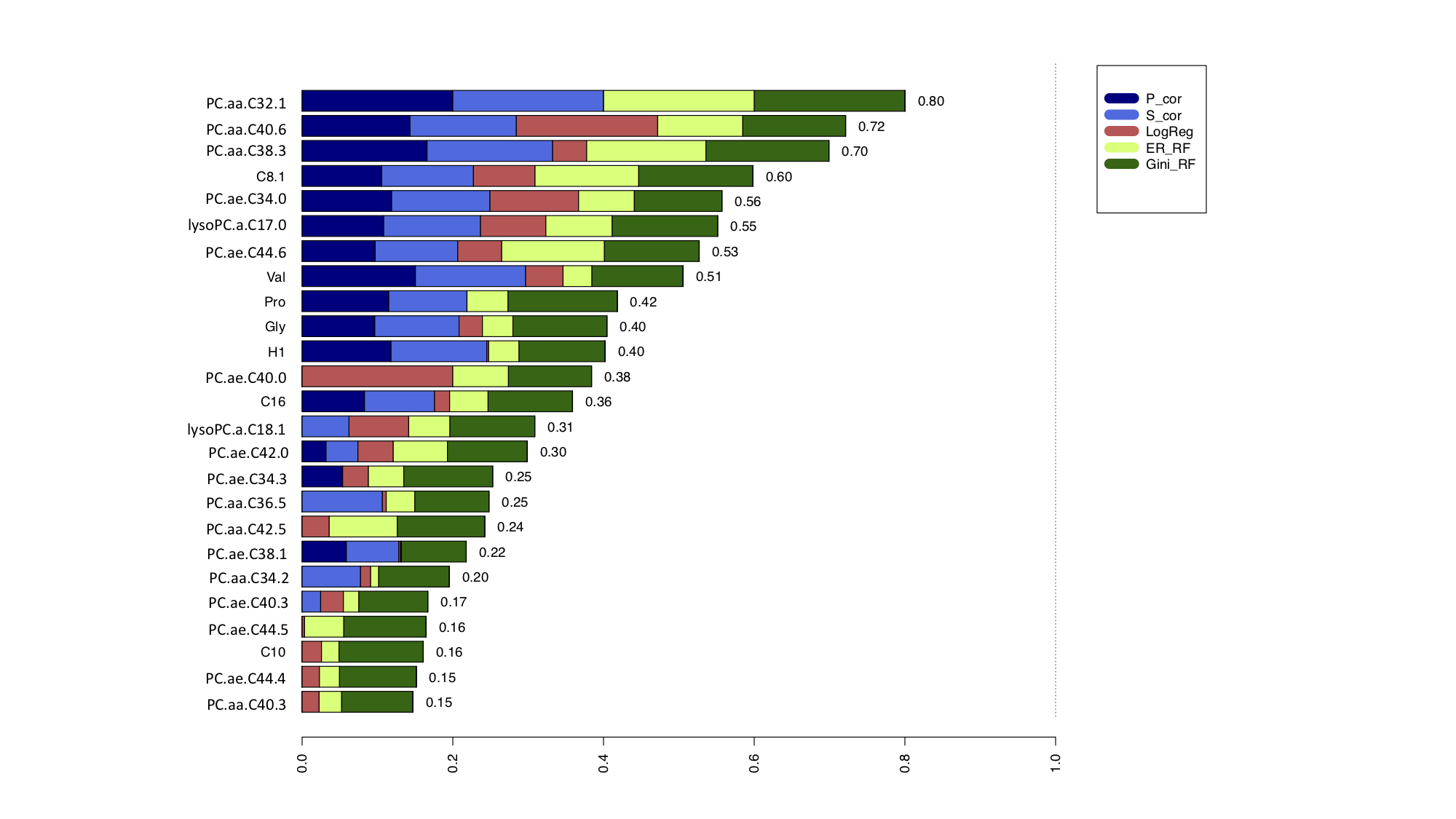

Supplement: S19 Fig — The y-axis shows the 25 targeted metabolomic variables ordered by importance value. The x-axis shows the cumulative importance values, calculated via an ensemble of feature selection methods including Spearman’s rank correlation test (S_cor), Pearson’s product moment correlation test (P_cor), beta-values of logistic regression (LogReg), error-rate-based variable importance measure (ER_RF), and Gini-index-based variable importance measure (Gini_RF). (TIFF) [file pmed.1003149.s019.tiff]

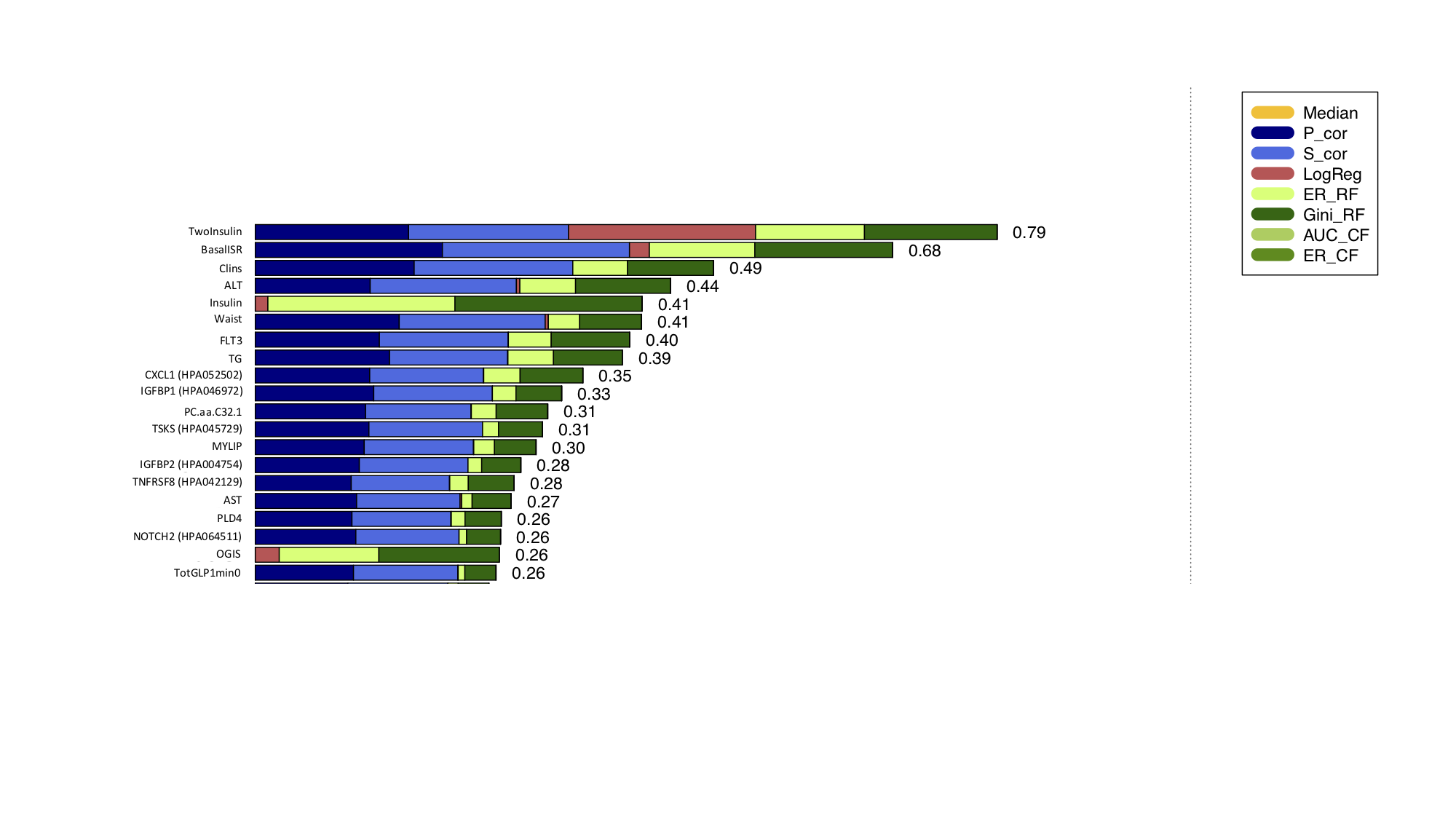

Supplement: S20 Fig — The y-axis shows the top 20 predictors in the model. The x-axis shows the cumulative importance values, calculated via an ensemble of feature selection methods including Spearman’s rank correlation test (S_cor), Pearson’s product moment correlation test (P_cor), beta-values of logistic regression (LogReg), error-rate-based variable importance measure (ER_RF), and Gini-index-based variable importance measure (Gini_RF). ALT, alanine transaminase; AST, aspartate transaminase; BasalISR, insulin secretion at the beginning of the OGTT/MMTT; Clins, mean insulin clearance during the OGTT/MMTT calculated as (mean insulin secretion)/(mean insulin concentration); Insulin, fasting insulin from venous plasma samples; OGIS, oral glucose insulin sensitivity index according to the method of Mari et al. [24]; TG, fasting triglycerides; TotGLP1min0, concentration of fasting total GLP-1 in plasma; TwoInsulin, 2-hour insulin after OGTT/MMTT. (TIFF) [file pmed.1003149.s020.tiff]
