# Supplementary material for: Predicting and elucidating the etiology of fatty liver disease: A machine learning modeling and validation study in the IMI DIRECT cohorts
Source: PLoS Med. 2020 Jun 19;17(6):e1003149. doi: 10.1371/journal.pmed.1003149 (PMC7304567; doi:10.1371/journal.pmed.1003149)
Supplement: S1 STROBE Checklist — (DOCX) [file pmed.1003149.s021.docx]

STROBE Statement: Checklist of items included in reports of *cohort studies* **(IMI DIRECT)**

| **Section/Topic** |  | Checklist Item | | Section and paragraph |
| --- | --- | --- | --- | --- |
| **Title and abstract** | |  |  |  |
| Title  Abstract | 1 | (*a*) Indicate the study’s design with a commonly used term in the title or the abstract | | Title |
|  |  | (*b*) Provide in the abstract an informative and balanced summary of what was done and what was found | | “Methods and Findings” |
| Introduction | | | | |
| Background/  rationale | 2 | Explain the scientific background and rationale for the investigation being reported | | Paragraphs 1-2 |
| Objectives | 3 | State specific objectives, including any prespecified hypotheses | | Paragraph 3 |
| Methods | | | | |
| Study design | 4 | Present key elements of study design early in the paper | | Section “*Participants (IMI DIRECT)”* |
| Setting | 5 | Describe the setting, locations, and relevant dates, including periods of recruitment, exposure, follow-up, and data collection | | Section “*Participants (IMI DIRECT)”* |
| Participants | 6 | (*a*) Give the eligibility criteria, and the sources and methods of selection of participants. Describe methods of follow-up | | Section “*Participants (IMI DIRECT)”* |
|  |  | (*b*) For matched studies, give matching criteria and number of exposed and unexposed | | Section “*Participants (IMI DIRECT)”* |
| Variables | 7 | Clearly define all outcomes, exposures, predictors, potential confounders, and effect modifiers. Give diagnostic criteria, if applicable | | Section “*Measures (IMI DIRECT)”* |
| Data sources/ measurement | 8* | For each variable of interest, give sources of data and details of methods of assessment (measurement). Describe comparability of assessment methods if there is more than one group | | Section “*Measures (IMI DIRECT)”*, Paragraphs 1-4 |
| Bias | 9 | Describe any efforts to address potential sources of bias | | Section “*Measures (IMI DIRECT)”,* Paragraphs 4-5 |
| Study size | 10 | Explain how the study size was arrived at | | Section “*Measures (IMI DIRECT)”,* Paragraphs 1-3 |
| Quantitative variables | 11 | Explain how quantitative variables were handled in the analyses. If applicable, describe which groupings were chosen and why | | Section “*Measures (IMI DIRECT)”,* Paragraph 4  Section “*Feature selection (IMI DIRECT)”* |
| Statistical methods | 12 | (*a*) Describe all statistical methods, including those used to control for confounding | | Section “*Feature selection (IMI DIRECT)”*  Section “*Model training and evaluation”* |
|  |  | (*b*) Describe any methods used to examine subgroups and interactions | | Section “*Model training and evaluation”* |
|  |  | (*c*) Explain how missing data were addressed | | Section “*Feature selection (IMI DIRECT)”*, Paragraph 1 |
|  |  | (*d*) If applicable, explain how loss to follow-up was addressed | | NA |
|  |  | (*e*) Describe any sensitivity analyses | | Section “*Model training and evaluation”*  Section “*Comparison with other fatty liver indices”*  Section “*External validation (UK Biobank cohort)”* |
| Results | | | |  |
| Participants | 13* | (a) Report numbers of individuals at each stage of study—eg numbers potentially eligible, examined for eligibility, confirmed eligible, included in the study, completing follow-up, and analysed | | Methods  Fig2  S5 Fig |
|  |  | (b) Give reasons for non-participation at each stage | | Section “*Additional proteomic and metabolomic analyses (models 15-18)”* |
|  |  | (c) Consider use of a flow diagram | | Methods  Fig2  S5 Fig |
| Descriptive data | 14* | (a) Give characteristics of study participants (eg demographic, clinical, social) and information on exposures and potential confounders | | Methods  Table 1  S2 Table |
|  |  | (b) Indicate number of participants with missing data for each variable of interest | | Methods  Fig2  S5 Fig |
|  |  | (c) Summarise follow-up time (eg, average and total amount) | | NA |
| Outcome data | 15* | Report numbers of outcome events or summary measures over time | | Methods  Fig2  S5 Fig |

| Main results | 16 | (*a*) Give unadjusted estimates and, if applicable, confounder-adjusted estimates and their precision (eg, 95% confidence interval). Make clear which confounders were adjusted for and why they were included | Section “*Clinical models (Models 1-3)*  Section “*Performance metrics”*  Section “*Omics models separately or in combination with clinical variables (models 5-14)”*  Section “*Additional proteomic and metabolomic analyses (models 15-18)”* |
| --- | --- | --- | --- |
|  |  | (*b*) Report category boundaries when continuous variables were categorized | Methods |
|  |  | (*c*) If relevant, consider translating estimates of relative risk into absolute risk for a meaningful time period | NA |
| Other analyses | 17 | Report other analyses done—eg analyses of subgroups and interactions, and sensitivity analyses | Section “*Validation in UK Biobank and IMI DIRECT”*  Section “*Comparison with other fatty liver indices”* |
| Discussion | | | |
| Key results | 18 | Summarise key results with reference to study objectives | Paragraph 1 |
| Limitations | 19 | Discuss limitations of the study, taking into account sources of potential bias or imprecision. Discuss both direction and magnitude of any potential bias | Paragraph 9-10 |
| Interpretation | 20 | Give a cautious overall interpretation of results considering objectives, limitations, multiplicity of analyses, results from similar studies, and other relevant evidence | Paragraphs 6-8 |
| Generalisability | 21 | Discuss the generalisability (external validity) of the study results | Paragraphs 11-13 |
| Other information | | | |
| Funding | 22 | Give the source of funding and the role of the funders for the present study and, if applicable, for the original study on which the present article is based | The work leading to this publication has received support from the Innovative Medicines Initiative Joint Undertaking under grant agreement n°115317 (DIRECT), resources of which are composed of financial contribution from the European Union's Seventh Framework Programme (FP7/2007-2013) and EFPIA companies’ in kind contribution. |

*Give information separately for exposed and unexposed groups.

**Note:** An Explanation and Elaboration article discusses each checklist item and gives methodological background and published examples of transparent reporting. The STROBE checklist is best used in conjunction with this article (freely available on the Web sites of PLoS Medicine at http://www.plosmedicine.org/, Annals of Internal Medicine at http://www.annals.org/, and Epidemiology at http://www.epidem.com/). Information on the STROBE Initiative is available at http://www.strobe-statement.org.
