# Supplementary material for: Predicting and elucidating the etiology of fatty liver disease: A machine learning modeling and validation study in the IMI DIRECT cohorts
Source: PLoS Med. 2020 Jun 19;17(6):e1003149. doi: 10.1371/journal.pmed.1003149 (PMC7304567; doi:10.1371/journal.pmed.1003149)
Supplement: S1 Text — (DOCX) [file pmed.1003149.s029.docx]

**S1 Text**.

*Transcriptomics*

For the study of transcriptomic profiles of participants at high risk of diabetes (WP2.1) and new

onset of diabetes (WP2.2) 3,094 samples of mRNA from whole blood samples were processed by University of Oxford and shipped to University of Geneva for RNA-sequencing. Concentration of mRNA per samples was assessed using the Qubit2.0 from Invitrogen. The quality of the samples was then assessed using the TapeStation Software (A.01.04) with an RNA Screen Tape from Agilent to check the mRNA quality on gel. 29 samples were discarded at this point due to low mRNA quality. The remaining samples were processed and sequencing libraries were prepared. Quality of the libraries was evaluated using Qubit and TapeStation using DNA1000 Screen Tape. One sample was discarded after library preparation due to low quality. The remaining samples (3,064) were placed in Flow cell PE using the cBOT system from Illumina. The samples were then sequenced on the Illumina HiSec2000 platform using 49 bp paired-end reads. Sequenced reads were mapped to the GRCh37 reference genome (2001) with GEM (Marco-Sola et al. 2012). Identification of samples mix-ups and labeling errors is possible when genotypes are available (tHoen et al. 2013). For each sample, we tested the heterozygous sites in DNA genotypes for expression of both alleles in the RNAseq data. Samples mix-ups or mislabeled show lower levels of expression of both alleles. Using the package matchASE from the suite QTL-tools (manuscript in preparation), we tested each expression profile (BAM files) against all imputed genotypes from DIRECT WP2 (release version October 2015) to identify the best matching expression-genotype pair. The analysis identified 201 samples with mismatch between expression and genotypes. Of those samples 137 could be corrected as we identified another genotype with a good match within the data set. One sample matched a genotype registered as not having mRNA sample. In these samples we are not certain whether genotype or RNAseq samples were swap with another individual. Use of the samples is not recommended in association with other phenotypes. Other 4 samples had low quality or were mixes of RNAseq samples and would not be identified with confidence. For 60 samples we would not find a suitable match between expression and the available genotypes. Gender identification in RNAseq compare expression levels of genes in the autosomal region for the chromosome Y and the expression of the XIST gene in the chromosome X. To confirm gender information, we compare the gender provided by clinical reports and the gender identified by genotype data with the gender identified from RNAseq data.

*Exploratory proteomics*

We performed antibody bead array assays for profiling of plasma proteins (Drobin K et al., 2013) using a selected set of 779 antibodies (targeting 385 proteins) availability from the Human Protein Atlas (HPA) (Uhlen et al., 2015). EDTA plasma samples from the four different DIRECT study centers were distributed across microtiter plates via a supervised randomization procedure. After sample randomization, the 96-well microtiter plates were stored at -80°C until further use. Plasma samples were thawed at 4°C and centrifuged for 10 min at 3,000 × g. Three microliters of each sample were diluted in 22 µl of 1x PBS using a liquid handler (SELMA, CyBio). As described earlier (Drobin K et al., 2013), samples were then biotinylated with NHS-biotin, the reaction was quenched using TRIS-HCl. Biotinylated samples were diluted in assay buffer, heated treated at 56°C for 30 min and then added the bead arrays prepared above. The magnetic beads were washed on the next day using a plate washer (EL406, Biotek). Fluorescent streptavidin was added for the detection of biotinylated proteins captured by bead-immobilized antibodies. The beads were analysed using the FlexMap 3D instrument (Luminex Corp.) operated by the xPONENT software version 4.2. The median fluorescence intensity (MFI) was used to represent the relative amount of target protein binding to each of the antibody-coupled bead identity.

The obtained data was evaluated based on intensity levels and three antibodies were excluded from further analysis eight samples were flagged that seemingly failed. Such samples were those 1) that had median values of MFIs ± 2 SD or below the median of control measurement without any sample (buffer only), and 2) that were identified as outliers using Robust PCA using ‘rrcov’ R package (version 1.4-3) (Hubert et al., 2005). The cutoff probability values in an outlier diagnostic plot were set to 0.001 for both score and orthogonal distances. The samples deviating beyond the cutoffs in both distance coordinates were classified as outliers, setting alpha, the proportional tolerance, to 0.9. The remaining data set was denoted as annotated.

*Targeted proteomics*

Samples from DIRECT study centers were manually randomization by a mix-shake-distribute procedure and placed into 96-well plates. All samples were analyzed at SciLifeLab in Stockholm using several different immunoassay platforms.

Proteins were measured in EDTA plasma using the Cardiometabolic, Cardiovascular II, Cardiovascular III, Development and Metabolism panels from Olink Proteomics AB (Uppsala, Sweden) according to the instructions for the Proximity Extension Assays (PEA) (PMID: 24755770). The obtained normalized expression values (NPX) values were obtained from Olink’s NPX manager software version 0.0.85.0. Magnetic bead-based assays were used for the analysis of FGF21 (SPRCUS627, MerckMillipore) and a panel consisting of For CXCL10, ICAM-1, IL1R-alpha, and RETN (LXSAHM, R&D Systems). The assays were performed according to the instructions and the instrumentation for liquid handling as introduced above. The beads were analyzed using the FlexMap 3D instrument (Luminex Corp.) operated by the xPONENT software version 4.2. The obtained MFI values were converted into concentration values using 5-parametric fitting. Plasma levels of IL1-beta and TNFR1-alpha were quantified in accordance with the instructions for the microfluidic ELISA assays (PMID: 27170460) from ProteinSimple.

Additional proteins were analyzed in randomized samples using the services from Myriad RBM (Myriad GmbH, Germany) and for hsCRP (MLM Medical Labs GmbH, Germany).

*Targeted metabolomics*

Plasma concentrations of 163 metabolites were determined using a targeted metabolomic approach with the Absolute*IDQ*^TM^ p150 kit (BIOCRATES Life Sciences AG, Innsbruck, Austria). After data export from Met*IDQ*, a first technical QC comprising analysis of peak shapes, retention times, and compound identity was performed. In a second QC step possible batch effects, study centre effects and effects of different phenotypes were investigated by using principal component analysis (PCA). The PCA of the data showed centre specific clusters. Further analysis showed phenotypic and technical (sample handling/ preparation/ collection/ storage and transport) differences between the study centres. Therefore, data were corrected for batches and study centres. Lower outliers were defined as samples with >33% of metabolite concentrations below 25% quantile – 1.5*IQR. Upper outliers were defined as samples with >33% of metabolite concentrations above 25% quantile + 1.5*IQR. Metabolite traits with too many zero concentration samples and NAs (>50%) were excluded (none). The Coefficient of Variation (CV) was calculated in reference samples for each metabolite over all plates. Metabolite traits with CV>0.25 were excluded. Metabolite traits with > 95% of samples below LOD were marked.

*Untargeted metabolomics*

Untargeted LC/MS-based techniques cover a broader spectrum of metabolites, in contrast to the targeted techniques where metabolites are limited to a predefined set of molecules. Non-targeted metabolomics data sets typically contain approx. 20-30% missing values, affecting more than 80% of the measured compounds. Missingness in the data can occur for several reasons. Firstly, a molecule might not be present at all in the sample. Secondly, missing values can be due to technical effects like, (i) metabolite concentrations are below the limit of detection (LOD), (ii) matrix or contamination effects hamper the quantification of a metabolite in a sample through co-eluting compounds due to altered ionization properties (signal suppression or enhancement), and (iii) challenges in computational processing of spectra, such peak picking and peak alignment (e.g. overlapping peaks). In order to understand how missingness seems within metabolon data, we compared the missing values of a metabolite between different run days and investigated the standard deviations of the run day missingness proportions across the metabolites. We further split the missing value distribution of metabolites as per the pathways, so that we know which pathways are informative or most relevant. We considered a sample to have a “low measurement” for a given metabolite if the sample's measurement was in the lower tail of the distribution of measurements for the metabolite. In such a case we called the metabolite "low". An outlier is a metabolite-sample pair where the distance of the log10-transformed metabolite measurement from the mean of the metabolite was greater than 4 times the standard deviation of the metabolite. Outliers were removed from the data set.

In the next QC step, we computed the coefficient of variation (CV) of measurements by run day for every metabolite. We then calculated the median CV over run days and used this as a measure of the variability of the measurement process. A median CV greater than 0.25 was considered as evidence that the measurement process was unable to yield reliable measurements for the given metabolite. In such cases we excluded the metabolite due to low quality. We also excluded a metabolite unless the CV could be computed for at least two run days. In particular, metabolites that had nothing but missing values in the reference plasma data were excluded. The number of metabolites were excluded later on due to a median CV over run days that is too high (> 0.25) or because there were so few non-missing measurements for a metabolite that its CV could only be computed for a single run day or not at all. In other words, we require a minimum of 2 run day CVs before we consider computing the median.

After these QC steps, missing values were imputed with the “multivariate imputation by chained equations” (MICE) method by using R package mice (version 2.2.5). We set the number of imputations to m=20 for all methods, assuming this to be a sufficient number to assure accuracy of the obtained estimates in a simulation study.
