# Supplementary material for: Predicting and elucidating the etiology of fatty liver disease: A machine learning modeling and validation study in the IMI DIRECT cohorts
Source: PLoS Med. 2020 Jun 19;17(6):e1003149. doi: 10.1371/journal.pmed.1003149 (PMC7304567; doi:10.1371/journal.pmed.1003149)
Supplement: Attachment — Submitted filename: Response to reviewers.docx [file pmed.1003149.s031.docx]

**Re: PMEDICINE-D-20-00136R1- Predicting and elucidating the etiology of fatty liver disease using a machine learning-based approach: an IMI DIRECT study**

**Requests from the editors:** Ref 3 raises some significant points and we would like to see all of these issues resolved in revisions.

**Response:** We did our best to address and resolve all the issues and concerns raised by the editors and reviewers. We hope that our revised manuscript has improved to the level of satisfaction.

**Other editorial points:**

**Comment:** Title - Please revise your title according to PLOS Medicine's style. Your title must be nondeclarative and not a question. It should begin with main concept if possible. "Effect of" should be used only if causality can be inferred, i.e., for an RCT. Please place the study design ("A randomized controlled trial," "A retrospective study," "A modelling study," etc.) in the subtitle (ie, after a colon). The study design should be a machine learning approach.

**Response:** We changed the title to “Predicting and elucidating the etiology of fatty liver disease: a machine learning modelling and validation study in the IMI DIRECT cohorts”

**Comment:** Abstract – (here and elsewhere including main text and tables) please ensure that p values are provided where 95%Cis are given.

**Response:** Confidence intervals are typically favored over P-values, as the former provides both an estimate of uncertainty and is informative of the statistical power of the test (PMID: 27209009). Moreover, in all instances, as the confidence intervals indicate, the P-values are highly statistically significant. Rather than add the highly statistically significant P-values to all of the CIs in the paper, we propose that we simple state at the first instance that “All of ROCAUCs are significant at P-value <0.00001.”(Line 342)

**Comment:** Please also include a sentence or two on the study’s limitations as the final part of the Methods and Findings section.

**Response:** We added the following text to the manuscript (Line 155):

“This study was undertaken in people of European ancestry and it is not known the extent to which the results will generalize to other ethnic groups. Moreover, the prediction is for a binary outcome (<5% or ≥5%) and neither quantifies liver fat volume nor elucidates the degree of liver damage (cirrhosis). These key limitations of the current work will be the focus of future research.”

**Comment:** Please also add summary demographic information to the abstract and please also mention the 2 cohorts from and where they are recruited from.

**Response:** We added the summary demographic information to the Abstract as requested (Line 7).

“We utilized the baseline data from the IMI DIRECT, a multicenter prospective cohort study of 3029 European ancestry adults recently diagnosed with T2D (n=795) or at high risk of developing the disease (n=2234).”

**Comment:** At this stage, we ask that you include a short, non-technical Author Summary of your research to make findings accessible to a wide audience that includes both scientists and non-scientists. The Author Summary should immediately follow the Abstract in your revised manuscript. This text is subject to editorial change and should be distinct from the scientific abstract. Please see our author guidelines for more information: <https://journals.plos.org/plosmedicine/s/revising-your-manuscript#loc-author-summary> 

**Response:** We have added the following text as Author Summary to the manuscript (Line 28):

**Why Was This Study Done?**

- About one in four adults have NAFLD globally, with considerable regional variations.
- The disease adversely affects energy homeostasis (particular blood glucose concentrations), blood detoxification, drug metabolism and food digestion.
- Although numerous non-invasive tests to detect NAFLD exist, these typically include inaccurate blood-marker tests or expensive imaging methods.
- The purpose of this work was to develop accurate non-invasive methods to predict fatty liver.
- We aimed to address an important gap in the clinical prediction of NAFLD and sought to elucidate the molecular mechanisms underlying the disease.

**What Did the Researchers Do and Find?**

- The analyses applied machine learning methods to data from the deep-phenotyped IMI DIRECT cohorts (n=1514) to identify sets of highly informative variables for the prediction of NAFLD. The criterion measure was liver fat quantified from MRI.
- We developed a total of 18 prediction models that range from very inexpensive models of modest accuracy to more expensive biochemistry- and/or omics-based models with high accuracy.
- We found that models using measures commonly collected in either clinical settings or in research studies proved adequate for the prediction of NAFLD.
- The addition of detailed omics data significantly improved the predictive utility of these models. We also found that of all omics markers, proteomic markers yielded the highest predictive accuracy when appropriately combined.

**What Do These Findings Mean?**

- We envisage that these new approaches to predicting fatty liver may be of clinical value when screening at risk populations for NAFLD.
- The identification of specific molecular features that underlie the development of NAFLD provides novel insights into the disease’s etiology, which may lead to the development of new treatments.
- These analyses have been performed in adults of European ancestry residing in northern Europe and it is unknown how well these findings will translate to people of other ancestries and exposed to environmental risk factors that contrast those of the present cohort.

**Comment:** Please use square brackets for refs in the main text instead of curved.

**Response:** We have now replaced the parentheses with brackets for the citations.

**Comment:** Please ensure that the study is reported according to the [STROBE] guideline (for the cohort aspect), and include the completed [STROBE or other] checklist as Supporting Information. When completing the checklist, please use section and paragraph numbers, rather than page numbers. Please add the following statement, or similar, to the Methods:

"This study is reported as per the Strengthening the Reporting of Observational Studies in Epidemiology (STROBE) guideline (S1 Checklist)." 
Please report your study according to the relevant guideline, which can be found here: <http://www.equator-network.org/>

--> transparent reporting of a multivariable prediction model for individual prognosis or diagnosis (TRIPOD) guideline (S1 Checklist).

**Response:** We have added the STROBE and TRIPOD to the Supporting Information and the statement to the manuscript (Line 307 and 469, respectively) as requested.

"This study is reported as per the Strengthening the Reporting of Observational Studies in Epidemiology (STROBE) guideline (S1 Checklist)."

"This study is reported as per the transparent reporting of a multivariable prediction model for individual prognosis or diagnosis (TRIPOD) guideline (S2 Checklist)."

**Comments from the reviewers:**

**Reviewer #1:** This study describes a NAFLD prediction model based on multi-omic and clinical data using machine learning. The topic is of great interest and the manuscript is overall well presented. 

**Response:** We thank the reviewer for the time and effort spent on this study.

**Reviewer #2:** This is a very interesting study with significant implications.

**Comment:** 1. The link between NAFLD and cardiovascular disease (CVD) should be briefly mentioned since it further highlights the importance of the early detection and treatment of NAFLD.

Useful refs: 
Targher G, Byrne CD, Lonardo A, Zoppini G, Barbui C. [Targher, 2016 #1184]. J Hepatol. 2016;65(3):589-600. 
Mahfood Haddad T, Hamdeh S, Kanmanthareddy A, Alla VM. Nonalcoholic fatty liver disease and the risk of clinical cardiovascular events: A systematic review and meta-analysis. Diabetes Metab Syndr. 2017;11 Suppl 1:S209-S216.

**Response:** We thank the reviewer for this suggestion and we have now added this information to the manuscript with the references (Line 74).

“Growing evidence also links the increased risk of cardiovascular events with NAFLD [6,7]. “

**Comment:** 2. Please comment on other relevant papers such as: 
Perakakis N, Polyzos SA, Yazdani A, Sala-Vila A, Kountouras J, Anastasilakis AD, Mantzoros CS. Non-invasive diagnosis of non-alcoholic steatohepatitis and fibrosis with the use of omics and supervised learning: A proof of concept study. Metabolism. 2019;101:154005. 
Katsiki N, Gastaldelli A, Mikhailidis DP. Predictive models with the use of omics and supervised machine learning to diagnose non-alcoholic fatty liver disease: A "non-invasive alternative" to liver biopsy? Metabolism. 2019;101:154010.

**Response:** We thank the reviewer for this suggestion and we have now cited these relevant papers and discussed them in the manuscript (Line 505).

“Machine-learning models based on lipidomic, glycomic and free fatty acid were also developed for the diagnosis of NASH and liver fibrosis [47, 48]”.

**Comment:** 3. What about the cost of equipments, stuff, measurements etc for this machine-learning approach? What about the complexity of the procedure? 
In other words, is it possible to perform this technique in daily clinical practice? 
How can we suggest that this approach is cost-effective? Are there any data?

**Response:** We thank the reviewer for this comment, but believe this may reflect a key misunderstanding of how this work could be translated into practice. That is, all machine learning analyses have been performed as part of the current work, the results from which are embedded into the fatty liver prediction website we provide a link to in the manuscript ([www.predictliverfat.org](http://www.predictliverfat.org)). The variables required to use this predication tool include some that are readily available in clinical practice (waist circumference, BMI, systolic and diastolic blood pressures, alcohol consumption, diabetes status, TG, fasting glucose (or HbA1c)) and others that could be easily obtained at relatively low cost (ALT, AST and fasting insulin levels). Thus, the cost connected with NAFLD prediction using the approaches proposed here are low and should be relatively easy to apply in clinical practice. We have not performed cost-effective analyses, as to do this well would require additional data on the costs and benefits of screening for NAFLD using the proposed models, which we do not have access to (as this would require a new prospective study).

**Reviewer #3:** This study built several machine-learning models for predicting non-alcoholic fatty liver disease based on clinical, genetic, transcriptomic, proteomic, and metabolomic data. The authors also built a nice website tool for clinicians to easily use their prediction models. 

**Comment:** 1. The authors tried to integrate multiple sources of data, however, the authors overlooked the innate differences of omics and clinical data in data type (discrete, continuous, categorical), scale and distribution. There is no data harmonization or normalization between different data source and simply putting them into one single prediction model and interpreting the importance ranking can be misleading.

In addition, many studies have revealed correlation between omics data, for example, genetic and transcriptomic data (i.e., eQTLs). However, the authors only applied LASSO within each omics data and did not discuss the potential collinearity issues between the selected omics features. One possible solution is that, instead of putting all features from multiple data sources in to one model, the authors can use boosting algorithms that ensemble the prediction model for each data source into one stronger prediction model.

“The authors tried to integrate multiple sources of data, however, the authors overlooked the innate differences of omics and clinical data in data type (discrete, continuous, categorical), scale and distribution.”

**Response:** We thank the reviewer for highlighting this very important point. To deal with different data types (numerical and categorical) in our analysis, we chose Random Forest as the training algorithm, which is known for its decent behavior in dealing with mixed data. We should also emphasize that almost all of the input variables in our models were continuous except for the alcohol consumption and diabetes status.

We have also inverse-normalized all different input data after correctness for the possible confounders prior to any analyses (Line 115, 153). Although utilizing the Random Forest as a non-parametric method also allows us to use the data as it is without need for any transformation.

“There is no data harmonization or normalization between different data source and simply putting them into one single prediction model and interpreting the importance ranking can be misleading.”

**Response:** We agree with the reviewer that by including correlated predictor variables the importance list could be misleading. We initial determined the variable importance in our analysis by a “permutation accuracy importance” measure using Random Forest in a very conservative cross-validated fashion, to further minimize this risk, we have now undertaken the EFS analysis which is an ensemble of feature selection methods to determine the normalized importance value of all features. With this function, we do not only rely on Random Forest for the importance ranking and we can build the cumulative importance values from different methods including Spearman’s rank correlation test, Pearson’s product moment correlation test, beta-Values of logistic regression, Error-rate-based variable importance measure and Gini-index-based variable importance measure.

The importance lists derived from EFS for different models were highly consistent with those derived from the Random Forest analysis. The minor inconsistencies between approaches is likely to reflect the ability of the Random Forest analysis to detect variables that interact with others, which the linear methods are not designed to detect. We have presented the variable importance results of EFS in S15-20 (see below) and we have also added the following texts to the manuscript”:

(Line 258):

“In addition, we have undertaken the EFS analysis which is an ensemble of feature selection methods to determine the normalized importance value of all features. With this approach, we do not only rely on Random Forest for the importance ranking and we can build the cumulative importance values from different methods including Spearman’s rank correlation test, Pearson’s product moment correlation test, beta-Values of logistic regression, Error-rate-based variable importance measure and Gini-index-based variable importance measure [30].”

(Line 430)

“Moreover, the importance list derived from EFS is shown in S20 Fig which is highly consistent with those derived from the Random Forest analysis. Rankings for the individual clinical and omics variables via “permutation accuracy importance” and EFS are presented in S8-19 Figs. The minor inconsistencies between approaches is likely to reflect the ability of the Random Forest analysis to detect variables that interact with others, which the linear methods are not designed to detect.”

“In addition, many studies have revealed correlation between omics data, for example, genetic and transcriptomic data (i.e., eQTLs). However, the authors only applied LASSO within each omics data and did not discuss the potential collinearity issues between the selected omics features. One possible solution is that, instead of putting all features from multiple data sources in to one model, the authors can use boosting algorithms that ensemble the prediction model for each data source into one stronger prediction model.”

**Response:** We thank the reviewer for the very interesting suggestion. We tried to use boosting algorithms and ensemble the prediction model for each data source to investigate if we can get a stronger prediction model as suggested. We trained a Stochastic Gradient Boosting algorithm for each data source separately and then applied a weighted averaging on the probabilities of observations. The optimal weighting was observed at 0.5 for the clinical data and 0.125 for each omic data layer (i.e., genetic, transcriptomic, exploratory proteomic and targeted metabolomic). The ensembled prediction model of omics and clinical datasets resulted in ROCAUC 0.83 (95% CI=0.78, 0.87) which is not materially different from the ROCAUC derived for the advanced model 14 (described in the Results), which includes all the omics and clinical features in a single model (ROCAUC of 0.84, 95% CI= 0.82, 0.86).

Our intention by including all features into the same model was to maximize their predictive power by leveraging interactions between features. We have now discussed this in the manuscript (Line 528).

“Our intention by including all features into the same model (model 14), was to maximize their predictive power by leveraging interactions between features. Moreover, we explored the value of boosting algorithms to ensemble the prediction model for each data source. The purpose of this was to enhance predictions. We trained a Stochastic Gradient Boosting algorithm for each data source separately and then applied a weighted averaging on the probabilities of observations. The optimal weighting was observed at 0.5 for the clinical data and 0.125 for each omic data layer (i.e., genetic, transcriptomic, exploratory proteomic and targeted metabolomic). The ensembled prediction model of omics and clinical datasets resulted in ROCAUC 0.83 (95% CI=0.78, 0.87) which is not materially different from the ROCAUC derived for the advanced model 14 (described in the Results), which includes all the omics and clinical features in a single model (ROCAUC of 0.84, 95% CI= 0.82, 0.86).”

We hope that we understood the Reviewer’s point correctly. If this is not what the Reviewer meant, could you please rephrase it and we would be happy to address it.

Other several minor aspects that the authors can improve:

**Comment:** 1. The flowchart is quite complicated. One suggestion is to build separate flowcharts for feature selection and model building, or at least put them in order so that the readers can clearly follow the steps.

**Response:** We have now separated the flowcharts for feature selection and model building as suggested. We have updated Fig2 and included details of the feature selection in S5 Fig.

**Fig 2.** Overview of the different stages involved in data processing and model training: Clinical (C), Genetic (G), Transcriptomic (T), Exploratory Proteomic (E-P), Targeted Proteomic (T-P), Targeted Metabolomic (T-M) and Untargeted Metabolomic (U-M). The green and blue dashed boxes illustrate the feature selection step the details of which can be found in S5 Fig.

**S5 Fig.** Details of the feature selection step for models 4-14 (blue box) and models 15-18 (green box)

**Comment:** 2. The QQ plot for the GWAS in the supplementary figure looks abnormal at the top. Please check what variables are used to conduct the GWAS and include all possible confounders.

**Response:** We are very grateful to the Reviewer for spotting this. We have now corrected our oversight and added BMI as the confounder to the GWAS analysis. We can clearly see that this improves the QQ-plot. We have changed the Manhattan plot and the following feature selection and model training analyses based on the new results (Figs 5, 6, S3, S4, S9 and Supporting Information (Excel file)). Furthermore, we have added an additional information text on filtering the genetic variants in our analysis. (Line216):

“We limited our analysis to genetic variants with a MAF > 1% and imputation quality score > 0.3.”

**S3 Fig.**

**S4 Fig.**

**Comment:** 3. The authors can expand details on what type of data standardization was done within each data source. Please clarify how "inverse normalized" was applied.

**Response:** Inverse normal transformation is a non-parametric method that replaces the data quantiles by quantiles from the standard normal distribution in order to reduce the impact of outliers and deviation from a normal distribution.

Inverse transformation of the variables can be obtained using the following command line in R:

transformedData <- qnorm((rank(Data)-0.5)/length(Data))

We have added the following text to the manuscript explaining the method (line 115).

“Inverse normal transformation is a non-parametric method that replaces the data quantiles by quantiles from the standard normal distribution in order to reduce the impact of outliers and deviation from a normal distribution.”

**Comment:** 4. While random forest is a useful machine learning model for classification, the authors can try at least one other method (e.g. XGBoost, support vector machines, k-nearest neighbor) and select the best performing models.

**Response:** Thank you for all these helpful suggestions. Some of the approaches the reviewer suggests were considered during the early phase of this project. We have now summarized all of these analyses (from Generalized Linear Model, Stochastic Gradient Boosting, support vector machines and k-nearest neighbor) in S6 Table (see below). As you will see, the results from the Random Forest analysis yield similar or better results compared with any of these other approaches.

We have added the following text to the manuscript (Line 557) and included the S6 Table in the Supporting Information.

“We have also considered several other machine learning approaches including Generalized Linear Model, Stochastic Gradient Boosting, support vector machines and k-nearest neighbor and the results from the Random Forest analysis yield similar or better results compared with any of these other approaches (see S6 Table).”

**S6 Table.** The ROCAUC results of each separate data set obtained from Random Forest (RF), Generalized Linear Model (GLM), Stochastic Gradient Boosting (GBM), support vector machines (SVM) and k-nearest neighbor (KNN) analyses in the cross-validated test data of IMI DIRECT combined cohorts.

|  | Clinical  (model 4) | Genetic  (model 5) | Transcriptomic (model 7) | | Proteomic (model 9) | Metabolomic  (model 11) |
| --- | --- | --- | --- | --- | --- | --- |
| RF | 0.79  (95% CI=0.76,0.81) | 0.67  (95% CI=0.65,0.70) | | 0.72  (95% CI=0.69,0.74) | 0.74  (95% CI=0.71,0.76) | 0.70  (95% CI=0.67,0.72) |
| GLM | 0.78  (95% CI=0.76, 0.80) | 0.63  (95% CI=0.60,0.66) | | 0.64  (95% CI=0.60, 0.65) | 0.71  (95% CI=0.68,0.73) | 0.70  (95% CI=0.66,0.72) |
| GBM | 0.78  (95% CI=0.76,0.80) | 0.66  (95% CI=0.63,0.69) | | 0.72  (95% CI=0.69,0.74) | 0.74  (95% CI=0.71,0.76) | 0.69  (95% CI=0.66,0.71) |
| SVM | 0.77  (95% CI=0.76,0.79) | 0.65  (95% CI=0.60,0.66) | | 0.67  (95% CI=0.64,0.69) | 0.70  (95% CI=0.67,0.72) | 0.71  (95% CI=0.68,0.73) |
| KNN | 0.73  (95% CI=0.70,0.75) | 0.60  (95% CI=0.57,0.63) | | 0.64  (95% CI=0.58,0.67) | 0.67  (95% CI=0.62,0.71) | 0.65  (95% CI=0.59,0.68) |

**Reviewer #4:** The paper is well written and the objectives are clear. Prediciton of NAFLD is an important aspect in for diagnostics, e.g., in case of the metabolic syndrome. I have only some minor comments: 

**Comment:** 1. Recently, Canbay et al. published a machine learning approach for the prediction of NAFLD with a similar AUC, with partly overlapping parameters, e.g., liver parameters. This paper should be discussed and compared to the findings in this study and the advantage of the new study should be clearly described.

**Response:** We thank the reviewer for this suggestion and we have now cited this relevant paper and discussed in the manuscript (Line 506).

“In a recent study of retrospectively collected obese individuals, EFS was undertaken to select the features out of the socio-demographic and serum parameters in order to predict presence or absence of NASH [48]”.

**Comment:** 2. The authors used mean decrease in accuracy as a measure of variable importance. However, this measure has been shown to be biased, see e.g., pubmed:17254353 or pubmed:23560875. An unbiased alternative could be the use of ensemble feature selection (EFS), see e.g., pubmed:28674556. I recommend to run an EFS selection, to compare the selected features with those from the RFs, and to discuss this at least in the discussion.

**Response:** We greatly thank the reviewer for the valuable suggestion. We applied the EFS to measure the important of variables. The importance lists derived from EFS for different models were highly consistent with those derived from the Random Forest analysis. The minor inconsistencies between approaches is likely to reflect the ability of the Random Forest analysis to detect variables that interact with others, which the linear methods are not designed to detect. We have presented the variable importance results of EFS in S15-20 (see below) and we have also added the following texts to the manuscript:

(Line 258) “In addition, we have undertaken the EFS analysis which is an ensemble of feature selection methods to determine the normalized importance value of all features. With this approach, we do not only rely on Random Forest for the importance ranking and we can build the cumulative importance values from different methods including Spearman’s rank correlation test, Pearson’s product moment correlation test, beta-Values of logistic regression, Error-rate-based variable importance measure and Gini-index-based variable importance measure [30].”

(Line430) “Moreover, the importance list derived from EFS is shown in S20 Fig which is highly consistent with those derived from the Random Forest analysis. Rankings for the individual clinical and omics variables via “permutation accuracy importance” and EFS are presented in S8-19 Figs. The minor inconsistencies between approaches is likely to reflect the ability of the Random Forest analysis to detect variables that interact with others, which the linear methods are not designed to detect.”

**S15 Fig.** Variable importance for the clinical model via EFS

**S16 Fig.** Variable importance for the genetic model via EFS

**S17 Fig.** Variable importance for the transcriptomic model via EFS

**S18 Fig.** Variable importance for the exploratory proteomic model via EFS

.

**S19 Fig.** Variable importance for the targeted metabolomic model via EFS

**S20 Fig.** Variable importance for the clinical plus multi-omics via EFS (1/2)

**S20 Fig.** Variable importance for the clinical plus multi-omics via EFS (2/2)
